# Mitochondrial ROS promote mitochondrial dysfunction and inflammation in ischemic acute kidney injury by disrupting TFAM-mediated mtDNA maintenance

**DOI:** 10.7150/thno.50905

**Published:** 2021-01-01

**Authors:** Meng Zhao, Yizhuo Wang, Ling Li, Shuyun Liu, Chengshi Wang, Yujia Yuan, Guang Yang, Younan Chen, Jingqiu Cheng, Yanrong Lu, Jingping Liu

**Affiliations:** 1 Key Laboratory of Transplant Engineering and Immunology, National Clinical Research Center for Geriatrics, Frontiers Science Center for Disease-related Molecular Network, West China Hospital, Sichuan University, Chengdu, China.; 2 Department of Nephrology, West China Hospital, Sichuan University, Chengdu, China.; 3 Animal Center, West China Hospital, Sichuan University, Chengdu, China.

**Keywords:** acute kidney injury, mitochondria, mtDNA, ROS, TFAM

## Abstract

**Aims:** Ischemia-reperfusion injury (IRI)-induced acute kidney injury (IRI-AKI) is characterized by elevated levels of reactive oxygen species (ROS), mitochondrial dysfunction, and inflammation, but the potential link among these features remains unclear. In this study, we aimed to investigate the specific role of mitochondrial ROS (mtROS) in initiating mitochondrial DNA (mtDNA) damage and inflammation during IRI-AKI.

**Methods:** The changes in renal function, mitochondrial function, and inflammation in IRI-AKI mice with or without mtROS inhibition were analyzed *in vivo*. The impact of mtROS on TFAM (mitochondrial transcription factor A), Lon protease, mtDNA, mitochondrial respiration, and cytokine release was analyzed in renal tubular cells *in vitro*. The effects of TFAM knockdown on mtDNA, mitochondrial function, and cytokine release were also analyzed *in vitro*. Finally, changes in TFAM and mtDNA nucleoids were measured in kidney samples from IRI-AKI mice and patients.

**Results:** Decreasing mtROS levels attenuated renal dysfunction, mitochondrial damage, and inflammation in IRI-AKI mice. Decreasing mtROS levels also reversed the decrease in TFAM levels and mtDNA copy number that occurs in HK2 cells under oxidative stress. mtROS reduced the abundance of mitochondrial TFAM in HK2 cells by suppressing its transcription and promoting Lon-mediated TFAM degradation. Silencing of TFAM abolished the Mito-Tempo (MT)-induced rescue of mitochondrial function and cytokine release in HK2 cells under oxidative stress. Loss of TFAM and mtDNA damage were found in kidneys from IRI-AKI mice and AKI patients.

**Conclusion:** mtROS can promote renal injury by suppressing TFAM-mediated mtDNA maintenance, resulting in decreased mitochondrial energy metabolism and increased cytokine release. TFAM defects may be a promising target for renal repair after IRI-AKI.

## Introduction

Acute kidney injury (AKI) is characterized by a rapid decline in renal function with high mortality and morbidity and is a serious health issue worldwide 1. AKI is increasing in incidence and can be caused by trauma, sepsis, surgery, or nephrotoxic drugs, and ischemia-reperfusion injury (IRI) is one of its leading causes 2. IRI disrupts the cellular redox balance and triggers excessive ROS production in the kidneys upon reperfusion, leading to a series of events that follow IRI-induced AKI (IRI-AKI) and include mitochondrial damage, energy depletion, tubular apoptosis and necrosis 3-5. Moreover, incomplete recovery from AKI can trigger renal fibrosis and thus increase the risk of chronic kidney disease (CKD) and end-stage renal failure (ESRD) 6-8. Currently, clinical interventions for AKI patients include renal replacement therapy (RRT) and nutritional supplementation. No effective therapy has been established, although many pharmacologic therapies such as diuretics, vasoactive drugs, and growth factors have been reported in animal models or preliminary clinical studies 9,10. Therefore, novel therapeutic targets for AKI are urgently needed.

The pathological mechanism of IRI-AKI is complicated, and oxidative stress is considered one of the primary factors leading to the condition 11,12. The overproduction of reactive oxygen species (ROS) contributes to multiple cellular processes related to renal injury, such as cytosolic calcium overload, energy depletion, apoptosis/necrosis, and inflammation 3. Increasing evidence indicates that the mitochondrion is a main source of intracellular ROS, and ~90% of ROS are generated in mitochondria 3. Mitochondrial ROS (mtROS) are primarily produced at complexes I and III of the electron transport chain (ETC) when electrons derived from either NADH or FADH_2_ react with O_2_ 13. The formation of mtROS due to proton leakage takes place under normal respiratory conditions but can be greatly enhanced in response to stress 14,15. The burst of ROS production that occurs after IRI-AKI has been shown to cause direct oxidative damage to mitochondrial proteins and lipids, thereby impairing mitochondrial bioenergetics by disrupting ETC function and increasing mitochondrial membrane permeability 16. Moreover, mtROS can also induce renal injury by activating proinflammatory signals such as Toll-like receptors (TLRs) and the NLRP3 inflammasome 17,18.

Although the adverse impact of mtROS on renal function has been demonstrated, the detailed mechanism by which ROS initiate mitochondrial dysfunction and inflammation during IRI-AKI is not completely understood. Recently, mitochondrial DNA (mtDNA) damage has emerged as a hallmark feature of AKI, and mtDNA depletion has been found both in preclinical models and in human patients with AKI 19. However, the link between mtROS and mtDNA damage during IRI-AKI remains unclear. mtDNA encodes 13 protein subunits of the ETC complexes and a set of transfer and ribosomal RNAs; thus, mitochondrial function is extremely dependent on functional mtDNA. mtDNA is a double-stranded circular DNA molecule that is present in multiple copies in the mitochondrial matrix and exists in condensed DNA-protein complexes known as nucleoids. The maintenance of mtDNA stability is primarily mediated by mitochondrial transcription factor A (TFAM), an essential mtDNA packaging protein that is required for mtDNA replication and transcription 20. Under pathological conditions, disruption of TFAM could lead to mtDNA depletion and deficient mitochondrial bioenergetics 21. Based on these findings, we speculated that ROS may disrupt the stability of mtDNA, but the detailed process still requires investigation.

In this study, we aimed to investigate the specific role of mtROS in mtDNA damage and inflammation during IRI-AKI. *In vivo* mouse IRI and *in vitro* cell hypoxia/reoxygenation (H/R) models were used to mimic the pathology of IRI-AKI. Changes in renal function, mitochondrial function, mtDNA maintenance, and TFAM expression were analyzed in the cell H/R and IRI-AKI mouse models with or without mtROS inhibition. The effects of mtROS on signaling pathways related to mtDNA maintenance and TFAM turnover were also analyzed.

## Methods

### Materials and reagents

The primary antibodies used in this study were purchased from the following sources: anti-Kim-1 (R&D Systems, USA); anti-TNF-α, anti-TOM20, anti-Bax (Cell Signaling Technology, Beverly, MA, USA); anti-8-OHdG, anti-dsDNA, anti-CD68 (Abcam, USA); anti-TFAM, anti-GAPDH, anti-Lon, anti-NGAL (ABclonal Biotech Co., Ltd., Cambridge, MA, USA); anti-p53, anti-ICAM, anti-ATP5a-1 (Proteintech, Rosemont, IL, USA). Mito-Tempo (MT, an mtROS scavenger) was purchased from Santa Cruz Biotechnology (CA, USA) and was dissolved in DMSO as a stock solution.

### Animal experiments

All animal experiments were approved by the Animal Care and Use Committee of West China Hospital, Sichuan University (No. 2018198A) and were conducted according to the National Institutes of Health Guide for the Care and Use of Laboratory Animals. Male C57BL/6 mice (20-25 g) were purchased from the Experimental Animal Center of Sichuan University (Chengdu, China). The animals were housed under standardized conditions with controlled temperature, humidity and 12-h cycles of light and darkness and provided with standard chow and tap water *ad libitum*. The mice were randomly divided into four groups (n = 6 per group): Control, IRI-AKI, IRI-AKI + MT, and IRI-AKI + vehicle. The mouse model of renal IRI injury used in this study was established as previously described 22. Briefly, the mice were anesthetized with 1% pentobarbital sodium (50 mg/kg, Merck, Darmstadt, Germany); mice with I/R injury received bilateral clamping of the renal pedicles for 30 min, and the mice in the control group received laparotomy only. To specifically inhibit the ROS burst in the kidney, an initial dose of 25 μL MT (5 μM in 0.05% DMSO in PBS) was directly injected into each kidney of the IRI-AKI mice using an insulin syringe after reperfusion; this was followed by daily intraperitoneal injection of MT (5 mg/kg, dissolved in 5% DMSO in PBS). The mice in the IRI-AKI alone group received vehicle at the same times and in the same volume as did the mice in the other groups using the same protocol. The body temperature of the animals was maintained at 37 °C during the surgery. On day 5 after surgery, the mice in each group were sacrificed by an overdose of anesthesia, and serum and renal samples were collected for further analysis.

### Biochemical analysis

Clinical biochemical analysis of the serum samples was performed on an automatic biochemistry analyzer (Cobas 6000, Roche Diagnostics, Switzerland); and the parameters, including creatinine (CREA) and blood urea nitrogen (UREAL), were measured using appropriate kits.

### Renal mtROS measurement

Renal tissues were stained with MitoSOX as previous described 23,24. Briefly, the fresh renal tissues removed from the mice were immediately frozen and cut into 5-µm-thick sections. The renal sections were incubated with MitoSOX Red dye (5 µM, Thermo Fisher Scientific, Sunnyvale, CA, USA) and Hoechst 33258 dye (10 μg/mL, Beyotime Biotechnology, Shanghai, China) in a dark, humidified container at 37 °C for 30 min, and images were acquired by fluorescence microscopy (Imager Z2, Zeiss, Germany).

### Renal mitochondrial morphology assay

Fresh renal tissues from the mice were fixed in 2.5% glutaraldehyde, dehydrated and embedded in EPON resin. Ultrathin sections prepared from the embedded tissues were stained with 5% uranyl acetate and lead citrate solution and observed by TEM (H-600, Hitachi, Ltd., Tokyo, Japan). Mitochondrial length/width ratio and average area were measured using ImageJ software (NIH, MD, USA) as previously described 24,25. In brief, ~15 mitochondria in each image were randomly selected, and the mitochondrial length and width were traced and measured using the Length parameter; mitochondrial area was measured using the Area parameter.

### Renal ATP measurement

ATP was measured using a bioluminescence assay kit (Beyotime Biotechnology, Shanghai, China). Briefly, the fresh kidney samples were lysed in the lysis buffer provided with the kit. The supernatant was collected by centrifugation at 12,000 rpm for 5 min at 4 °C. The concentration of ATP present in the samples was determined by mixing 20 μL of the supernatant with 100 μL of luciferase reagent; the luciferase present in the reagent catalyzes the production of luminescence from ATP and luciferin. The luminescence of each sample was measured on a microplate luminometer (Synergy Mx, BioTek Instruments Inc., Winooski, VT, USA). A standard curve of ATP was prepared using a series of standards of known concentrations; the measured ATP is presented as nmol/mg of protein.

### Immunofluorescence (IF) staining

Cells and frozen renal tissue sections were fixed with 4% paraformaldehyde in PBS for 10 min at room temperature, washed with PBS, and permeabilized with 0.3% Triton X-100 for 10 min. After blocking in 1% BSA for 30 min, the cells were immunolabeled with primary antibodies (anti-Kim-1 at 1:150 dilution; anti-TNF-α at 1:100 dilution; anti-dsDNA at 1:400 dilution; anti-TOM20 at 1:100 dilution; or anti-TFAM at 1:50 dilution) overnight at 4 °C followed by incubation with FITC- or TRITC-conjugated secondary antibody (1:200) for 1 h at 37 °C. Nuclei were visualized by staining with DAPI for 5 min at room temperature. Digital images of the sections were captured using a fluorescence microscope (Nikon, N-STORM & A1, Tokyo, Japan), and the Pearson's correlation coefficient between the TFAM and dsDNA results was analyzed using Image-Pro Plus.

### Renal histopathology

Renal tissues were fixed in 10% formalin and embedded in paraffin, and 5-µm sections of the embedded kidneys were stained with hematoxylin and eosin (H&E). Cell apoptosis was measured by TUNEL staining (Promega, USA) of the frozen renal sections according to the manufacturer's protocol. For immunohistochemical (IHC) staining, the renal sections were incubated with primary antibodies including anti-TFAM (1:50), anti-8-OHdG (1:100), and anti-CD68 (1:100) overnight at 4 °C and then with HRP-conjugated secondary antibodies (Millipore, MA, USA) and the DAB substrate. Micrographs of the stained sections were captured by light microscopy (Zeiss Imager A2, Germany) and quantified using ImageJ (NIH, Bethesda, MD, USA).

### RNA isolation and real-time PCR

Total RNA was extracted from renal tissue and cells using Trizol (Gibco, Life Technologies, CA, USA) and reverse-transcribed into cDNA using an iScript cDNA synthesis kit (Bio-Rad, USA). The primers used in this study are listed in Table S1. Real-time polymerase chain reaction (real-time PCR) was performed using SYBR Green PCR mix (Vazyme Biotech, Nanjing, China) in a real-time PCR apparatus (Bio-Rad, CA, USA). The data were analyzed using Bio-Rad CFX Manager software, and relative changes in mRNA levels were calculated by the delta-delta Ct method with GAPDH as the internal reference gene.

### Cell culture and treatment

The human renal proximal tubule epithelial cell line HK2 was cultured in DME/F12 medium (HyClone, USA) supplemented with 10% fetal bovine serum (FBS, Gibco, CA, USA), 100 U/mL penicillin, and 100 µg/mL streptomycin in a humidified atmosphere at 37 °C with 5% CO_2_. Oxidative stress of the cells was induced by treatment with tert-butyl hydroperoxide (t-BHP, 80 μM, Sigma-Aldrich, Taufkirchen, Germany) or hypoxia (≤ 1% O_2_, 5% CO_2_, 94% N_2_ for 24 h)/reoxygenation (21% O_2_, 5% CO_2_, 74% N_2_ for 2 h); excessive mtROS in cells were eliminated by treatment of the cells with MT (25 nM). To determine the roles of various sources and types of ROS, the cells were treated with specific ROS inhibitors, including catalase (CAT, 10 μM), GKT (an NADPH oxidase 4 (Nox4) inhibitor, 5 µM, CSN Pharm, Shanghai, China), apocynin (APO, an NADPH oxidase inhibitor, 10 µM, CSN Pharm, Shanghai, China), and 1400W (an inducible nitric oxide synthase (iNOS) inhibitor, 50 µM, CSN Pharm, Shanghai, China). The activity of Lon protease was inhibited using bortezomib (Borte, 5 µM, CSN Pharm, Shanghai, China) as previously described 26.

### Small RNA (siRNA) interference

HK2 cells were cultured to ~50-60% confluence in culture medium containing no penicillin or streptomycin. TFAM siRNA (sense: GAGGGAACUUCCUGAUUCATT; antisense: UGAAUCAGGAAGUUCCCUCTT), Lon siRNA (sense: CCGAGAACAAGAAGGACUUTT; antisense: AAGUCCUUCUUGUUCUCGGTT), and the negative control siRNA were purchased from GenePharma Biotechnology (Shanghai, China). HK2 cells were transfected with siRNA using Lipo6000 (Beyotime Biotechnology, Shanghai, China) according to the manufacturer's instructions.

### Cell viability

Cells were seeded in 96-well plates and treated with the indicated agents. CCK-8 (Dojindo, Kumamoto, Japan) solution was added to each well, and the plates were incubated at 37 °C for 2 h. The absorbance at 450 nm was then measured using a microplate reader (BioTek Instruments Inc, USA). The viability of the cells in the experimental groups was normalized to that of the cells in the control group.

### Measurement of intracellular ROS

The level of ROS was measured by flow cytometry and confocal microscopy. For the flow cytometry analysis, cells were incubated with MitoSOX (2 µM, Thermo Fisher Scientific, Sunnyvale, CA, USA) for 30 min, washed twice with PBS and analyzed in a flow cytometer (Beckman, USA). For the fluorescence microscopy assay, HK2 cells were incubated with DCFH-DA (10 μM, Beyotime Biotechnology, Shanghai, China) or MitoSOX (5 µM) for 30 min at 37 °C. The stained cells were then washed with PBS and observed using a fluorescence microscope (Olympus, Japan).

### Oxygen consumption rate (OCR) assay

The mitochondrial oxygen consumption rate (OCR) was measured in a Seahorse XF-24 Flux Analyzer (Seahorse Biosciences, Agilent, USA) using the Mito Stress Test (MST) Kit. Cells (5 × 10^4^ per well) were plated in quadruplicate in XF-24 extracellular flux assay plates in 500 μL XF base medium equilibrated to a pH of 7.4. After incubation of the plates at 37 °C for 2 h to allow the cells to adhere, the medium was replaced with MST buffer. The test compounds were added in the following order: oligomycin (1 μM), FCCP (1 μM), and rotenone/antimycin A (both 0.5 μM). The values obtained in each measurement for quadruplicate wells were averaged and are displayed as the OCR (pmol O_2_/min).

### Cellular mitochondrial morphology assessment

Mitochondrial morphology in live cells was observed by staining with MitoTracker Deep Red (100 nM, Thermo Fisher Scientific, Sunnyvale, CA, USA) followed by confocal microscopy (Nikon A1, Nikon Corporation, Japan). Mitochondrial length was analyzed using Image-Pro Plus software (Media Cybernetics, Inc., USA). The FF (form factor) was defined as (perimeter^2^/4 π area). The AR (aspect ratio) was defined as the ratio between the major and minor axes of an ellipse equivalent in size to the mitochondrion. The AR represents the mitochondrial length, and the FF indicates the mitochondrial complexity 27. FF and AR in images were analyzed using ImageJ software as previously described 28.

### Chemotaxis assay

The macrophage chemotaxis assay was performed using cell culture inserts containing a 3.0-μm-pore polyethylene terephthalate membrane (Corning, USA). Conditioned medium (500 μL) harvested from HK2 cells cultured under various conditions was added to each well of a 24-well plate (lower compartment), and inserts were placed in each well along with Raw 264.7 macrophages (5 × 10^5^ cells in 100 μL assay medium; upper compartment). After incubation of the plate at 37 °C for 24 h, the cells that had migrated from the upper to the lower compartment were collected and counted as previously described 29. The migrated cells were fixed with 4% paraformaldehyde at room temperature for 15 min and stained with 0.1% crystal violet (Sigma). Images of the stained cells were captured using a microscope, and the cell numbers were calculated using ImageJ.

### DNA isolation and mtDNA copy number assay

Relative levels of mitochondrial DNA (mtDNA copy number) were determined by qPCR as previously described 30, 31. Briefly, total DNA was extracted from cells or from renal tissues using a Universal Genomic DNA Kit (CW2298S, CWBIO, Beijing, China), and 10 ng of the DNA was used for qPCR analysis. For human cells, the mitochondrial *ND1* gene (mtND1) was used to measure mtDNA copy number and was normalized to nuclear beta-2 microglobulin (*B2M*) 30. For mouse tissues, the mitochondrially encoded cytochrome c oxidase subunit 2 (*COX2*) was used to measure mtDNA copy number and was normalized to the nuclear ribosomal protein s18 (*rps18*) 31. The sequences of the primers used in the mtDNA assay are shown in Table S2.

### Western blot

Renal tissues and cells were lysed in radioimmunoprecipitation assay (RIPA) buffer supplemented with protease inhibitors (Calbiochem, CA, USA) and phosphatase inhibitors (Calbiochem). The protein concentration was determined using a BCA protein assay kit (CWBIO, China). Equal amounts of protein were subjected to electrophoresis on 12% sodium dodecyl sulfate-polyacrylamide gels (SDS-PAGE) and then transferred to polyvinylidene difluoride membranes (PVDF, Merck Millipore). The membranes were blocked with 5% nonfat milk and incubated with one of the following primary antibodies: anti-Bax (#2772), anti-TFAM (A1926), anti-ATP5a-1 (14676-1-AP), anti-TOM20 (#42406), anti-Lon (A4293), anti-p53 (10442-1-AP), anti-ICAM-1 (10831-1-AP), or anti-GAPDH (AC002) overnight at 4 °C. After washing with PBST, the PVDF membranes were incubated with HRP-conjugated secondary antibody at 37 °C for 1 h. The protein bands on the PVDF membranes were observed using an enhanced chemiluminescence kit (Millipore, USA) and quantified using ImageJ.

### Human samples and IF staining

Human renal tissues that had sustained renal injury were obtained from renal biopsies of AKI patients. Normal kidney tissues obtained from surgical nephrectomy of renal carcinoma patients were used as controls. The process of human sample collection was performed in accordance with the guidelines established by the hospital ethics committee, and informed consent was obtained from the patients according to the Declaration of Helsinki. For IF staining, frozen renal sections were stained with primary antibodies (anti-TOM20, 1:100; anti-NGAL, 1:100; anti-dsDNA, 1:400; anti-TFAM, 1:50) overnight at 4 °C followed by incubation with an FITC- or TRITC-conjugated secondary antibody (1:200) for 1 h at 37 °C. Nuclei were visualized by staining with DAPI for 5 min at room temperature. Digital images were captured using a fluorescence microscope (Nikon, N-STORM & A1, Tokyo, Japan).

### Statistical analysis

All data are presented as the mean ± SD and were analyzed using SPSS software (version 11.5, IBM Corporation, USA) with one-way ANOVA or Student's *t*-test; *p* < 0.05 was considered indicative of a significant difference.

## Results

### mtROS promoted renal dysfunction, inflammation, and mitochondrial damage in IRI-AKI mice

**Figure 1:**
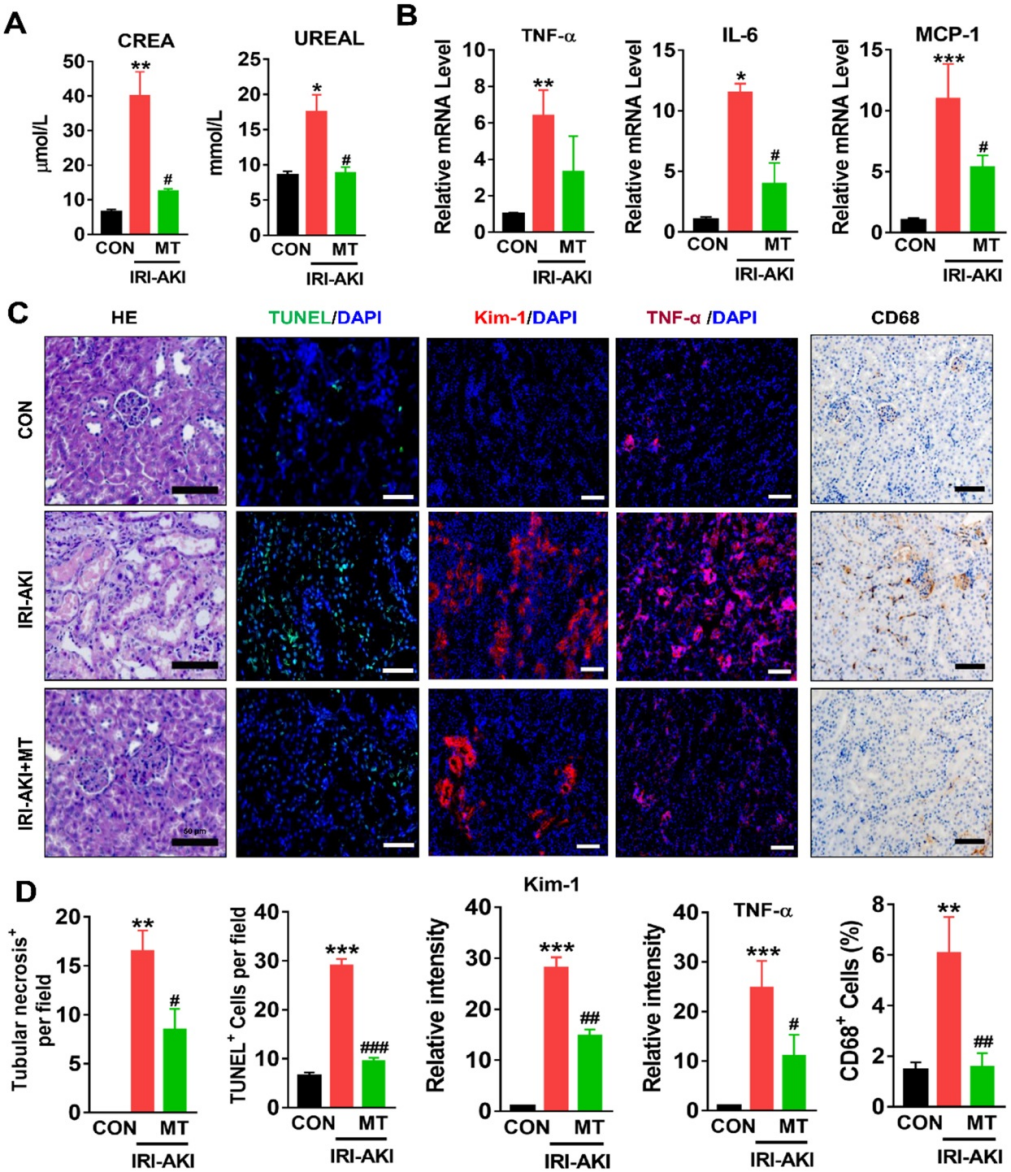
** mtROS promoted renal dysfunction and inflammation in IRI-AKI mice.** (A) Serum CREA and UREAL concentrations in mice in different groups on day 5 after IRI-AKI (n = 6). (B) Real-time PCR analysis of TNF-α, IL-6, and MCP-1 mRNA in the kidneys of mice in different groups on day 5 after IRI-AKI (n = 6). (C) Representative micrographs showing renal H&E staining (scale bar = 50 µm), TUNEL staining (scale bar = 100 µm), Kim-1 staining (scale bar = 100 µm), TNF-α IF staining (scale bar = 100 µm), and CD68 IHC staining (scale bar = 50 µm) in the kidneys of mice on day 5 after IRI-AKI. (D) Quantitative analysis of necrotic tubules, TUNEL^+^ apoptotic cells, Kim-1 expression, TNF-α expression, and CD68^+^ cell number in the kidneys of mice in different groups (n = 6). *p < 0.05 *vs.* CON group; **p < 0.01 *vs.* CON group; ***p < 0.001 *vs.* CON group; **^#^***p* < 0.05 *vs.* IRI-AKI group;**^ ##^***p* < 0.01 *vs.* IRI-AKI group;**^ ###^***p* < 0.001 *vs.* IRI-AKI group.

**Figure 2:**
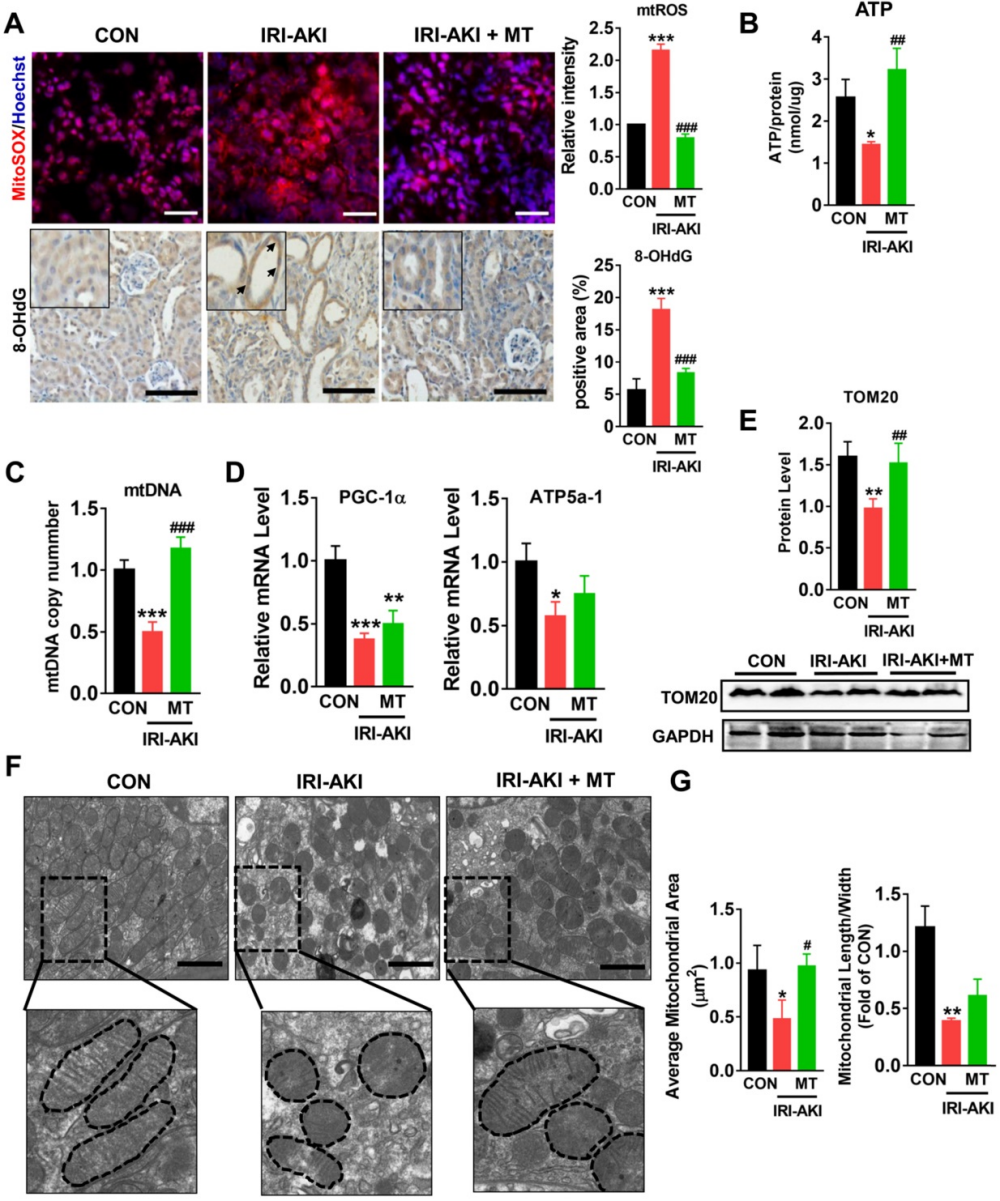
**mtROS promoted renal mitochondrial dysfunction in IRI-AKI mice.** (A) Representative micrographs showing MitoSOX (scale bar = 50 µm) and 8-OHdG staining (scale bar = 50 µm) in the renal cortex and quantitative analysis of mtROS and 8-OHdG levels (n = 6). (B) Measurement of ATP levels in the kidneys of mice on day 5 after IRI-AKI (n = 6). (C) Measurement of mtDNA copy number levels in the kidneys on day 5 after IRI-AKI (n = 6). (D) Real-time PCR analysis of PGC-1-α and ATP5a-1 mRNA levels in the kidneys on day 5 after IRI-AKI (n = 6). (E) Western blotting for TOM20 protein in the kidneys and quantitative analysis of TOM20 protein expression. (F) Representative TEM images of mitochondria in the renal tubules of mice (scale bar = 2 µm). (G) Quantification of mitochondrial area and the ratio of mitochondrial length to width detected by TEM (n = 6). *p < 0.05 *vs.* CON group; **p < 0.01 *vs.* CON group; ***p < 0.001 *vs.* CON group; **^#^***p* < 0.05 *vs.* IRI-AKI group;**^ ##^***p* < 0.01 *vs.* IRI-AKI group;**^ ###^***p* < 0.001 *vs.* IRI-AKI group.

Compared with control mice, IRI-AKI mice showed higher levels of serum CREA/UREAL and greater numbers of renal histological lesions, including cast formation, vacuolization, and extensive tubular necrosis, as well as elevated levels of kidney injury molecule-1 (Kim-1) and TUNEL^+^ apoptotic cells in the kidneys. The degree of renal inflammatory response as indicated by the expression of cytokines (TNF-α, IL-6, and MCP-1) and infiltration of the tubulointerstitial compartment by CD68^+^ macrophages was also upregulated in mice with IRI-AKI (Figure 1A-D). In contrast, inhibition of mtROS production with MT significantly ameliorated renal dysfunction, as indicated by decreased levels of serum CREA/UREAL, renal Kim-1, tubular apoptosis/necrosis, and renal inflammatory response in MT-treated IRI-AKI mice compared with untreated IRI-AKI mice (Figure 1A-D). Moreover, the kidneys of IRI-AKI mice exhibited severe mitochondrial damage, as evidenced by increased mtROS levels and decreases in ATP level, PGC-1α/ATP5a-1/TOM20 expression, mitochondrial area, and mitochondrial length/width ratio compared to the controls (Figure 2A-G). Mitochondrial lesions such as mitochondrial swelling and fragmentation, disruption of membrane integrity, and broken or absent cristae were observed in the kidneys of the IRI-AKI mice (Figure 2F). The degree of renal oxidative stress, as indicated by 8-OHdG 32, was also increased in the IRI-AKI mice compared to that in the control mice (Figure 2A). Conversely, MT treatment significantly reduced the levels of mtROS, 8-OHdG, and mitochondrial fragmentation and restored the ATP production and expression of mitochondrial proteins in the kidneys of the IRI-AKI mice (Figure 2A-G). However, treatment with the vehicle alone did not affect the degree of renal injury, inflammation, or mitochondrial dysfunction in IRI-AKI mice (Figure S1).

### mtROS-induced mitochondrial impairment and proinflammatory cytokine release in renal tubular cells (TECs)

**Figure 3:**
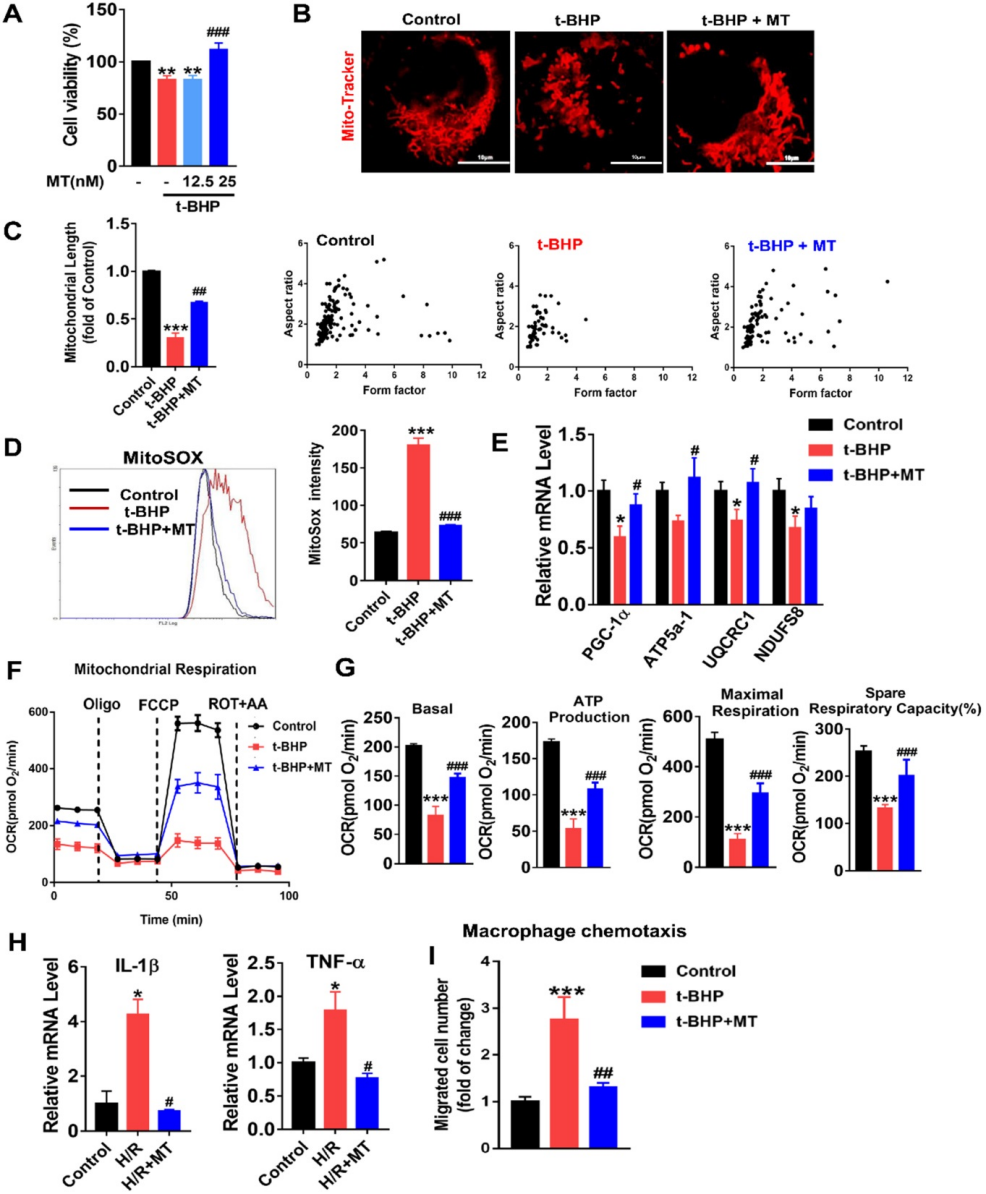
**mtROS-induced mitochondrial dysfunction and cytokine release in HK2 cells.** (A) Cell viability was determined by the CCK8 assay (n = 3, **p < 0.01 *vs.* Control group, **^###^***p* < 0.001 *vs.* t-BHP group). (B) Representative micrographs showing MitoTracker staining in HK2 cells (scale bar = 10 µm) and (C) quantification of mitochondrial length. (D) mtROS were measured by flow cytometry after MitoSOX staining. (E) Real-time PCR analysis of PGC-1α, UQCRC1, NDUFS8, and ATP5a-1 mRNA levels in HK2 cells. (F) Measurement of mitochondrial oxygen consumption ratio (OCR) in HK2 cells. (G) Basal respiration, maximal respiration, ATP production, and spare respiratory capacity in HK2 cells (n = 3; ***p < 0.001 *vs.* Control group; **^###^***p* < 0.001 *vs.* t-BHP group). (H) Real-time PCR analysis of IL-1β and TNF-α mRNA levels in HK2 cells (n = 3; *p < 0.05 *vs.* Control group; **^#^***p* < 0.05 *vs.* H/R group). (I) Relative migration of RAW264.7 cells in response to conditioned medium from HK2 cells (n = 3; ***p < 0.001 *vs.* Control group; **^##^***p* < 0.01 *vs.* t-BHP group).

To mimic oxidative stress in IRI-AKI, HK2 cells were stimulated with H/R or t-BHP (a ROS inducer). The elevated mtROS in the HK2 cells induced by H/R or t-BHP was effectively reduced by MT treatment (Figure S2A-C). Oxidative stress decreased the viability of the HK2 cells, while MT treatment restored the cells' viability in a dose-dependent manner (Figure 3A). Moreover, oxidative stress induced by t-BHP or H/R resulted in an increase in mitochondrial fragmentation (Figure 3B, Figure S4A) as well as a reduction in the expression of mitochondrial biogenesis-related genes such as ATP5a-1, PGC-1α, NDUFS8, and UQCRC1 in the HK2 cells (Figure 3E, Figure S4B). The overall mitochondrial respiratory capacity of the HK2 cells was also inhibited by t-BHP treatment (Figure 3F-G). In contrast, MT treatment markedly attenuated the mitochondrial lesions caused by mtROS in HK2 cells treated with t-BHP or H/R (Figure 3B-G, Figure S4). In addition, the increased levels of proinflammatory cytokine (IL-1β, TNF-α) gene expression and macrophage chemotaxis induced in the HK2 cells by oxidative stress were suppressed by the MT treatment (Figure 3H-I). Again, treatment with the vehicle alone had no influence on the expression of cytokines or proapoptotic factors in HK2 cells under stress conditions (Figure S7).

### mtROS suppressed TFAM transcription and promoted Lon-mediated TFAM degradation in TECs

**Figure 4:**
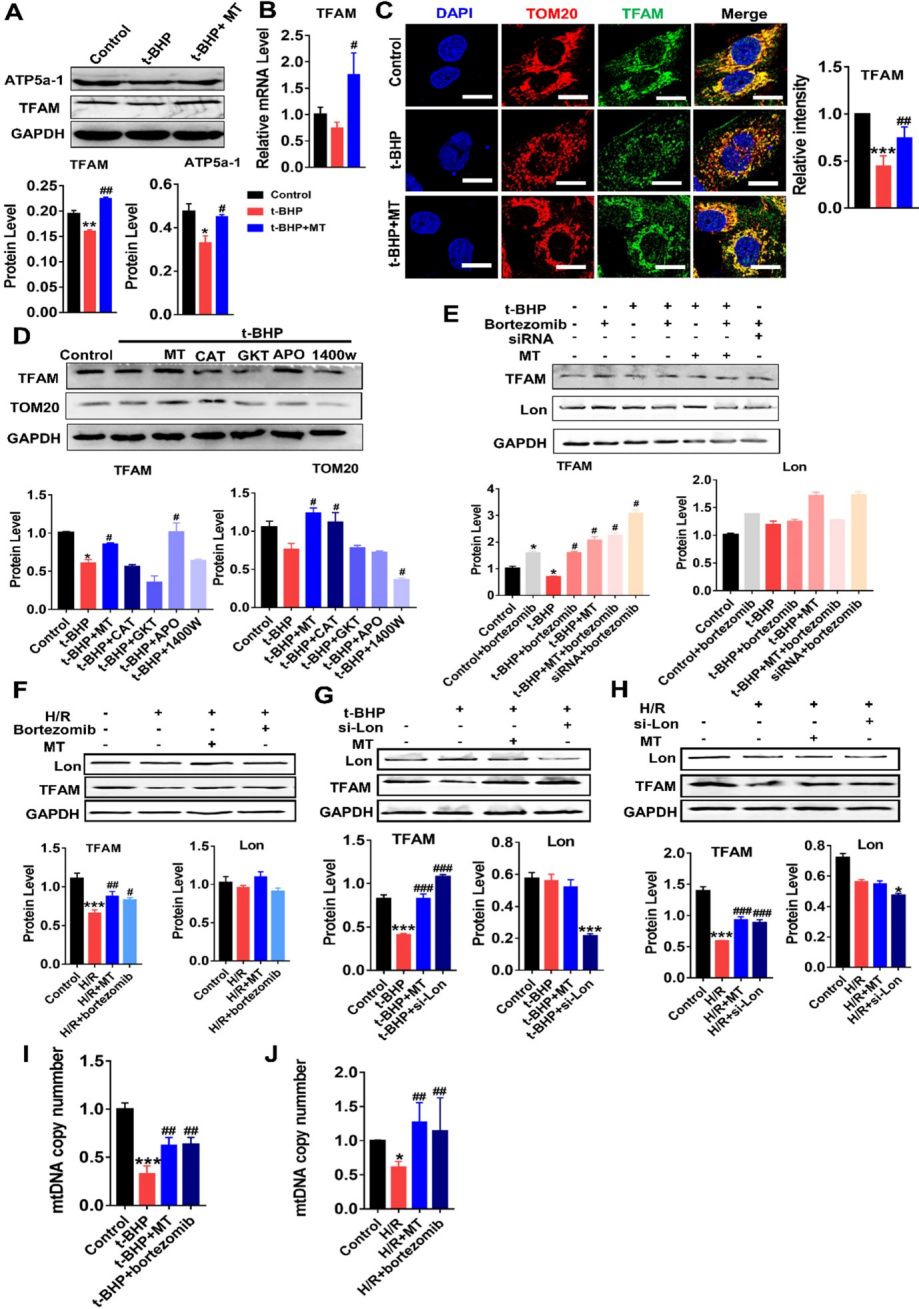
** mtROS suppressed TFAM transcription and enhanced TFAM degradation in HK2 cells.** (A) Western blotting for TFAM and ATP5a1 proteins in HK2 cells and quantitative analysis of protein expression (n = 3; *p < 0.05 *vs.* Control group, **p < 0.01 *vs.* Control group; **^#^***p* < 0.05 *vs.* t-BHP group, **^##^***p* < 0.01 *vs.* t-BHP group). (B) Real-time PCR analysis of TFAM mRNA levels in HK2 cells. (C) Double-IF staining of TOM20 (red) and TFAM (green) in HK2 cells (scale bar = 10 µm) and quantitative analysis of TFAM expression (***p < 0.001 *vs.* Control group; **^##^***p* < 0.01 *vs.* t-BHP group). (D) Western blotting for TFAM and TOM20 proteins in HK2 cells after various treatments and quantitative analysis of protein expression (n = 3; *p < 0.05 *vs.* Control group; **^#^***p* < 0.05 *vs.* t-BHP group). (E) Western blotting for TFAM and Lon proteins in HK2 cells treated with t-BHP and various other agents (n = 3; *p < 0.05 *vs.* Control group; **^#^***p* < 0.05 *vs.* t-BHP group). (F) Western blotting of TFAM and Lon proteins in HK2 cells under H/R with or without bortezomib treatment (n = 3; ***p < 0.001 *vs.* Control group; **^#^***p* < 0.05 *vs.* H/R group, **^##^***p* < 0.01 *vs.* H/R group). (G) Western blotting of TFAM and Lon proteins in HK2 cells treated with t-BHP with or without si-Lon treatment (n = 3; ***p < 0.001 *vs.* Control group; **^###^***p* < 0.001 *vs.* t-BHP group). (H) Western blotting of TFAM and Lon protein and quantitative analysis of protein expression in HK2 cells under H/R with or without si-Lon treatment (n = 3; ***p < 0.001 *vs.* Control group; **^###^***p* < 0.001 *vs.* H/R group). (I) mtDNA copy number in HK2 cells treated with t-BHP and various other agents (n = 3; ***p < 0.001 *vs.* Control group; **^##^***p* < 0.01 *vs.* t-BHP group). (J) mtDNA copy number in HK2 cells under H/R with various treatments (n = 3; *p < 0.05 *vs.* Control group; **^##^***p* < 0.01 *vs.* H/R group).

We next determined whether TFAM expression in renal cells is affected by oxidative stress. Indeed, the protein levels of TFAM and ATP5α-1 in HK2 cells were downregulated by t-BHP, whereas their protein levels partially recovered after MT treatment (Figure 4A). The mRNA level of TFAM was suppressed by mtROS and was also restored by MT treatment (Figure 4B). The majority of the TFAM colocalized with the mitochondrial marker TOM20, but t-BHP stimulation decreased the abundance of TFAM in the mitochondria, whereas the mitochondrial TFAM level recovered after MT treatment (Figure 4C). Similarly, H/R conditions also reduced the TFAM protein level (Figure 4F), especially the amount of TFAM in mitochondria (Figure S5), while the decrease in TFAM protein under H/R conditions was also reversed by MT treatment (Figure 4F and Figure S5). To determine the impact of different ROS sources and types on TFAM protein, HK2 cells under oxidative stress were treated with various ROS inhibitors. Interestingly, the CCK-8 assay results showed that the viability of HK2 cells under oxidative stress was improved by treatment with MT and other ROS inhibitors (Figure S3A), suggesting that oxidative stress-induced cell damage is a complex event that affects both the cytosolic and mitochondrial systems, since the CCK-8 assay results reflect the cellular redox status and can be influenced by a variety of cytosolic and mitochondrial oxidases 33. However, the decreased TFAM protein level induced by oxidative stress was only restored by treatment with MT and APO (an NADPH oxidase inhibitor), whereas the other inhibitors failed to rescue TFAM protein expression (Figure 4D). Treatment with MT, but not treatment with APO, restored the mitochondrial mass (as indicated by TOM20 expression) in HK2 cells exposed to t-BHP (Figure 4D).

The impact of mtROS on TFAM degradation was also analyzed. Interestingly, the protein level of Lon in HK2 cells was unchanged in response to the ROS induced by t-BHP or H/R, but its protease activity was upregulated by mtROS, as indicated by the elevated amount of TFAM protein in cells stimulated by t-BHP or H/R plus bortezomib (a Lon protease inhibitor) compared to that in cells stimulated by t-BHP or H/R alone (Figure 4E-F, S3B-C). There was no difference in TFAM protein expression in the MT and MT plus bortezomib groups after t-BHP treatment (Figure 4E). The TFAM level, which decreased after treatment with si-TFAM, also recovered after bortezomib treatment (Figure 4E). p53 protein was used as a marker of the activity of bortezomib (Figure S3B-C). Treatment with the vehicle alone did not affect TFAM expression in HK2 cells under oxidative stress (Figure S7A-B). To confirm the specific role of Lon in TFAM degradation, HK2 cells with Lon knockdown were generated using siRNA (Figure S6D). Treatment with si-Lon alone was sufficient to increase TFAM protein levels under normal culture conditions (Figure S6D). Moreover, treatment with si-Lon restored the expression of TFAM protein in HK2 cells exposed to t-BHP or H/R, and it showed a rescue effect on the TFAM protein level similar to that of MT treatment (Figure 4G-H). As a result, the reduction in mtDNA copy number in HK2 cells exposed to t-BHP or H/R was reversed by treatment with MT or bortezomib (Figure 4I-J).

### Loss of TFAM caused by mtROS led to mitochondrial dysfunction in TECs

**Figure 5:**
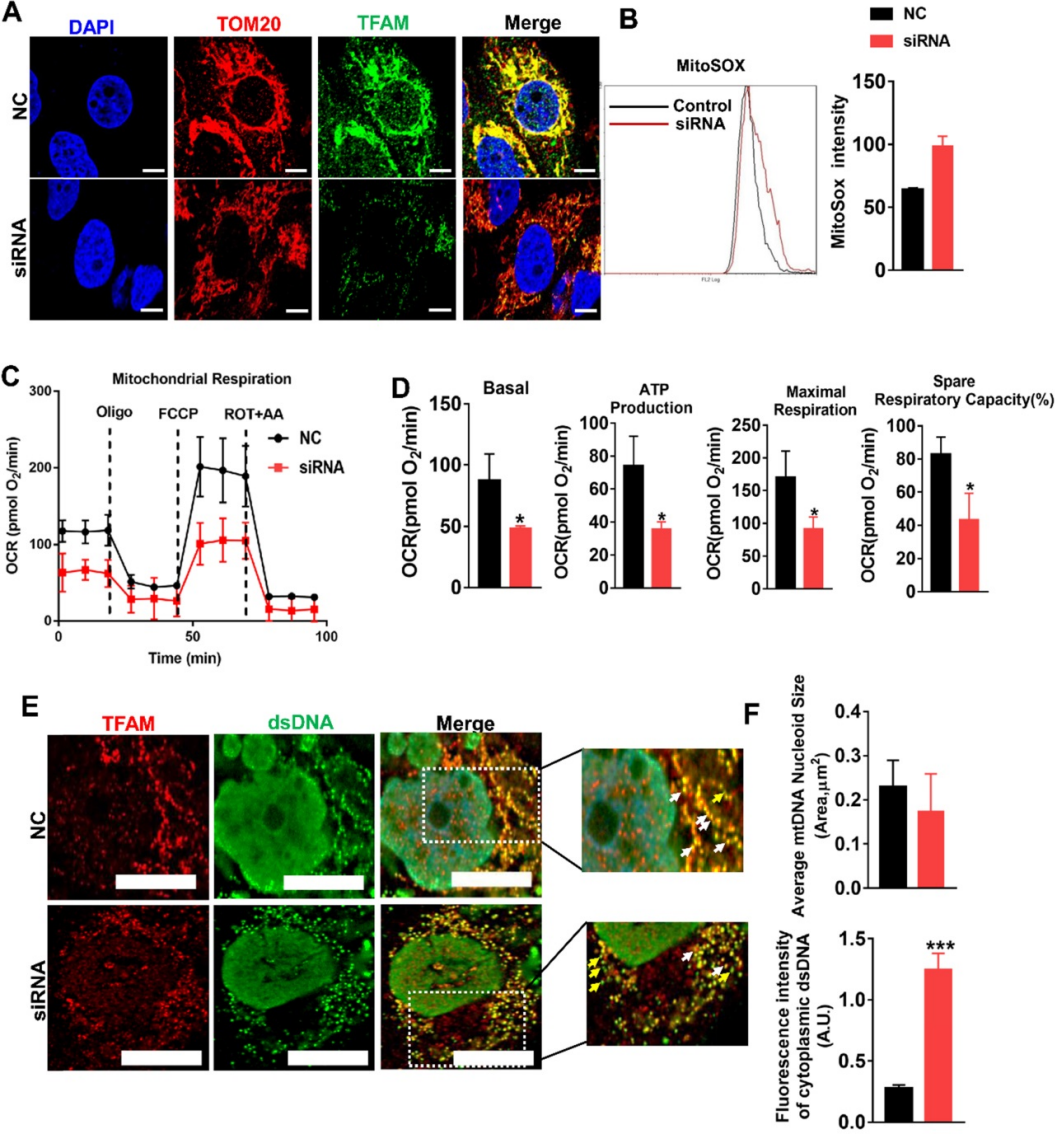
** Loss of TFAM was sufficient to induce mitochondrial dysfunction in HK2 cells.** (A) Double-IF staining of TOM20 (red) and TFAM (green) in HK2 cells (scale bar = 10 µm). HK2 cells were transfected with normal control siRNA (NC) and TFAM siRNA (siRNA). (B) The intensity of mtROS was determined by flow cytometry. HK2 cells were treated with control siRNA or TFAM siRNA (n = 3). (C) Measurement of mitochondrial oxygen consumption ratio (OCR) in HK2 cells. (D) Basal respiration, ATP production respiration, maximal respiration, and spare respiratory capacity in HK2 cells (n = 3; *p < 0.05 *vs.* Control group). (E) Double-IF staining of TFAM (red) and dsDNA (green) in HK2 cells (scale bar = 10 µm). (F) Average size of mtDNA nucleoids and cytoplasmic dsDNA (dsDNA without colocalization of TFAM) in HK2 cells detected by IF staining (n = 20; ***p < 0.001 *vs.* Control group).

**Figure 6:**
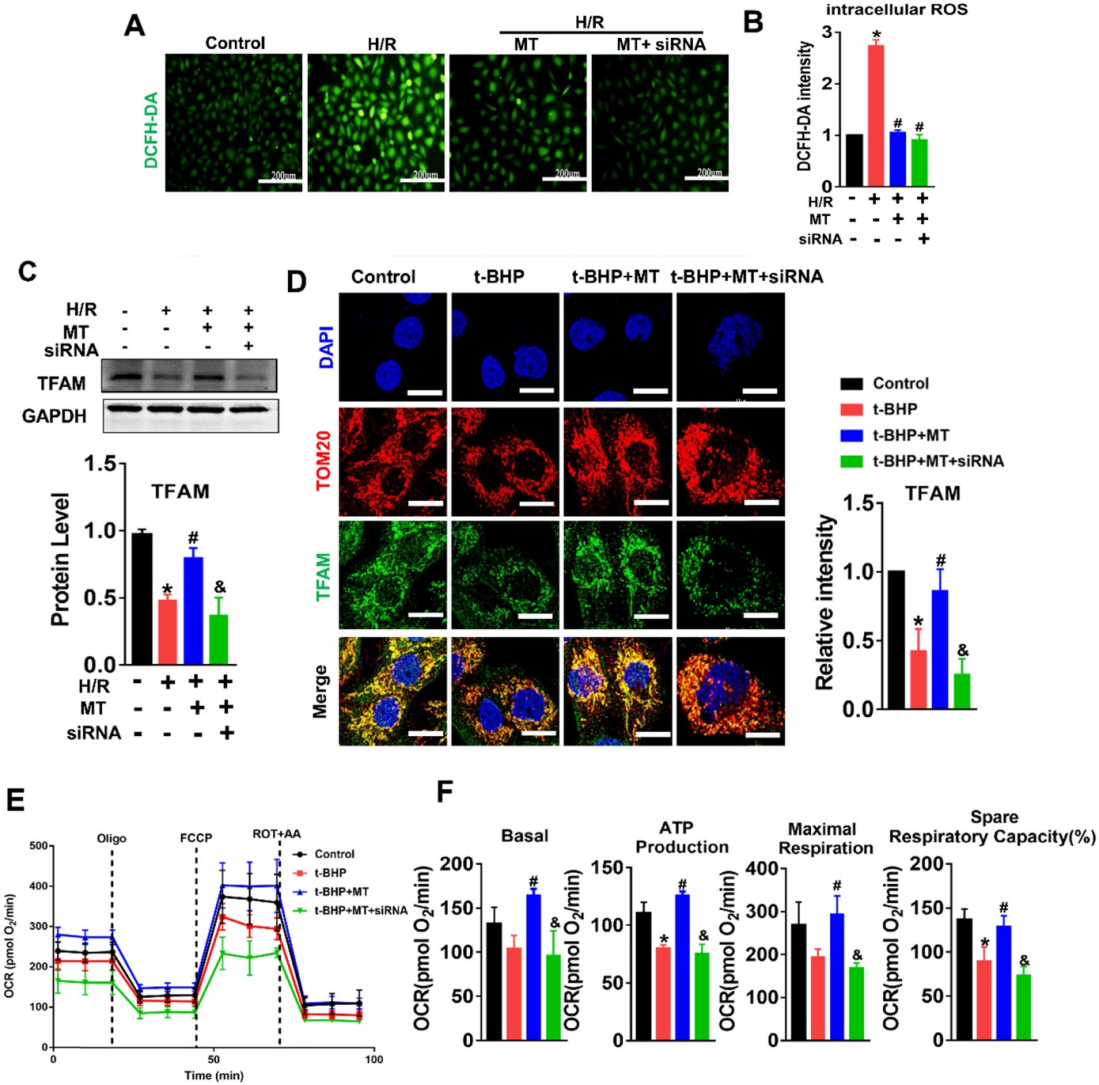
**mtROS impaired mitochondrial function by suppressing TFAM in HK2 cells.** (A) DCFH-DA fluorescence staining of HK2 cells (scale bar = 200 µm). (B) Quantitative analysis of intracellular ROS in HK2 cells (n = 3; *p < 0.05 *vs.* Control group; **^#^***p* < 0.05 *vs.* H/R group). (C) Western blotting of TFAM protein in HK2 cells and quantitative analysis of protein expression (n = 3; *p < 0.05 *vs.* Control group; **^#^***p* < 0.05 *vs.* H/R group; **^&^***P* < 0.05 *vs*. MT group). (D) Double-IF staining of TOM20 (red) and TFAM (green) in HK2 cells and quantitative analysis of TFAM expression (scale bar = 10 µm). (E) Measurement of mitochondrial oxygen consumption ratio (OCR) in HK2 cells after various treatments. (F) Basal respiration, ATP production, maximal respiration, and spare respiratory capacity in HK2 cells (n = 3; *p < 0.05 *vs.* Control group; **^#^***p* < 0.05 *vs.* t-BHP group; **^&^***P* < 0.05 *vs*. MT group).

Since our experiments revealed mtROS-induced TFAM defects in TECs, we sought to explore the effects of TFAM deficiency on mtDNA and mitochondrial function. TFAM knockdown cells were generated, and the reduction in TFAM levels was confirmed by qPCR and western blotting (Figure S6A-B). Knockdown of TFAM led to a reduction in the abundance of TFAM in the mitochondria (Figure 5A) and to increased levels of mtROS in HK2 cells under normal culture conditions (Figure 5B). The loss of TFAM led to decreased mitochondrial respiratory capacity in the HK2 cells, as indicated by the lower levels of basal and maximal respiration, ATP production, and spare respiratory capacity observed in the TFAM knockdown cells compared to the cells in the control group (Figure 5C-D). Moreover, the colocalization of TFAM and dsDNA (double-stranded DNA) was reduced by si-TFAM, suggesting that the stability of mtDNA nucleoids was also impaired (Figure 5E-F), considering that it has been reported that both naked DNA and free TFAM are unstable in mitochondria 34,35. Next, we determined the role of TFAM in mtROS-induced mitochondrial damage in TECs. The elevation in ROS levels induced by H/R or H/R plus si-TFAM was suppressed by MT (Figure 6A-B), but the reduced expression of TFAM in cells under stress was not reversed by MT treatment in the presence of si-TFAM (Figure 6C). TFAM knockdown also abolished the ability of MT to restore the protein level of TFAM in the mitochondria of HK2 cells experiencing oxidative stress induced by t-BHP (Figure 6D), and this finding was further validated in HK2 cells under H/R conditions (Figure S5). As a result, MT failed to attenuate the mtROS-induced mitochondrial respiratory defects in HK2 cells when TFAM was knocked down (Figure 6E-F).

### mtROS-induced mtDNA instability and cytokine release by suppressing TFAM in TECs

**Figure 7:**
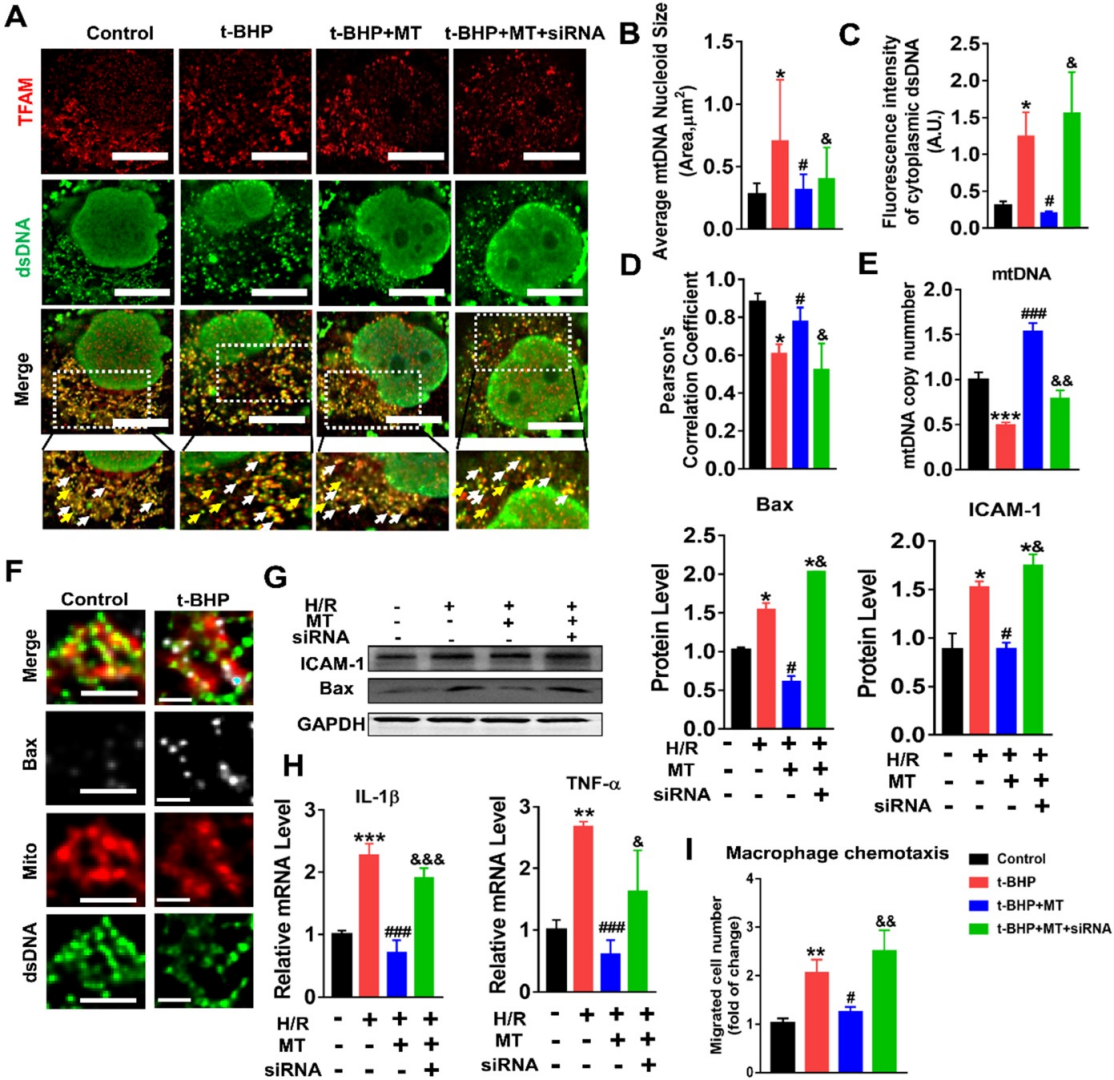
**Loss of TFAM-induced mtDNA instability and cytokine production in HK2 cells.** (A) Double-IF staining of TFAM (red) and dsDNA (green) in HK2 cells (scale bar = 10 µm). (B) Average size of mtDNA nucleoids in HK2 cells (n = 20; *p < 0.05 *vs.* Control group; **^#^***p* < 0.05 *vs.* t-BHP group; **^&^***p* < 0.05 *vs*. MT group). (C) Quantification of cytoplasmic dsDNA in HK2 cells (n = 6; ***p < 0.001 *vs.* Control group; **^###^***p* < 0.001 *vs.* t-BHP group; **^&&^***p* < 0.01 *vs*. MT group). (D) Pearson correlation coefficient between TFAM and dsDNA in HK2 cells in different groups (*p < 0.05 *vs.* Control group; **^#^**p < 0.05 *vs.* t-BHP group; **^&^***p* < 0.05 *vs*. MT group). (E) mtDNA copy number in HK2 cells (n = 3; *p < 0.05 *vs.* Control group; **^#^***p* < 0.05 *vs.* t-BHP group; **^&^***p* < 0.05 *vs*. MT group). (F) IF staining of dsDNA (green), Bax (white), and mitochondria (Mito; red) in HK2 cells (scale bar = 2.5 µm). (G) Western blotting of ICAM-1 and Bax proteins in HK2 cells and quantitative analysis of protein expression (n = 3; *p < 0.05 *vs.* Control group; **^#^***p* < 0.05 *vs.* H/R group; **^&^***p* < 0.05 *vs*. MT group). (H) Real-time PCR analysis of IL-1β and TNF-α mRNA levels in HK2 cells (n = 3; **p < 0.01 *vs.* Control group; ***p < 0.001 *vs.* Control group;**^ ###^***p* < 0.001 *vs.* H/R group; **^&^***p* < 0.05 *vs*. MT group, **^&&&^***p* < 0.001 *vs*. MT group). (I) Relative migration of RAW264.7 cells in response to conditioned medium from HK2 cells (n = 3; **p < 0.01 *vs.* Control group; **^#^***p* < 0.05 *vs.* H/R group; **^&&^***p* < 0.01 *vs*. MT group).

The impact of mtROS on mtDNA stability was evaluated, and mtDNA nucleoids were visualized using TFAM and dsDNA costaining. The mtDNA nucleoids, as indicated by the colocalization of TFAM and dsDNA, were evenly distributed as a network in normal control HK2 cells (Figure 7A, white arrows). Under oxidative stress conditions, the HK2 cells displayed aberrant mtDNA packaging and reduced mtDNA copy number (Figure 7A-E), as indicated by the presence of enlarged nucleoid clusters arranged in a fragmented network, increased amounts of leaked mtDNA (dsDNA without colocalization of TFAM, yellow arrows), and a decrease in the TFAM/dsDNA colocalization coefficient compared to the control (Figure 7A-D). Conversely, inhibition of mtROS by MT treatment attenuated the aberrant mtDNA packaging and restored TFAM/dsDNA colocalization and mtDNA copy number in HK2 cells under oxidative stress, but the beneficial effect of MT was eliminated when TFAM was knocked down (Figure 7A-E). Similarly, H/R induced mtDNA nucleoid damage in the HK2 cells, while the aberrant mtDNA packaging was partially reversed by MT treatment, but the rescue effect of MT was also limited by treatment with the TFAM siRNA (Figure S8). In addition, we observed that Bax protein colocalized with mitochondria (indicated by the MitoTracker) and that it formed macropore-like structures in which mtDNA appeared within the pores (Figure 7F). The upregulation of Bax in HK2 cells under oxidative stress was suppressed by MT treatment, while the inhibitory effect of MT on Bax was abolished when TFAM was knocked down (Figure 7G). Oxidative stress also induced cytokine (IL-1β, TNF-α, and ICAM-1) expression (Figure 7G-H) and macrophage chemotaxis (Figure 7I) in HK2 cells, and these adverse effects were reduced by MT treatment. However, knockdown of TFAM enhanced cytokine expression and macrophage chemotaxis in HK2 cells even in the presence of MT (Figure 7G-I).

### mtROS impaired TFAM and mtDNA homeostasis in the kidneys of IRI-AKI mice

**Figure 8:**
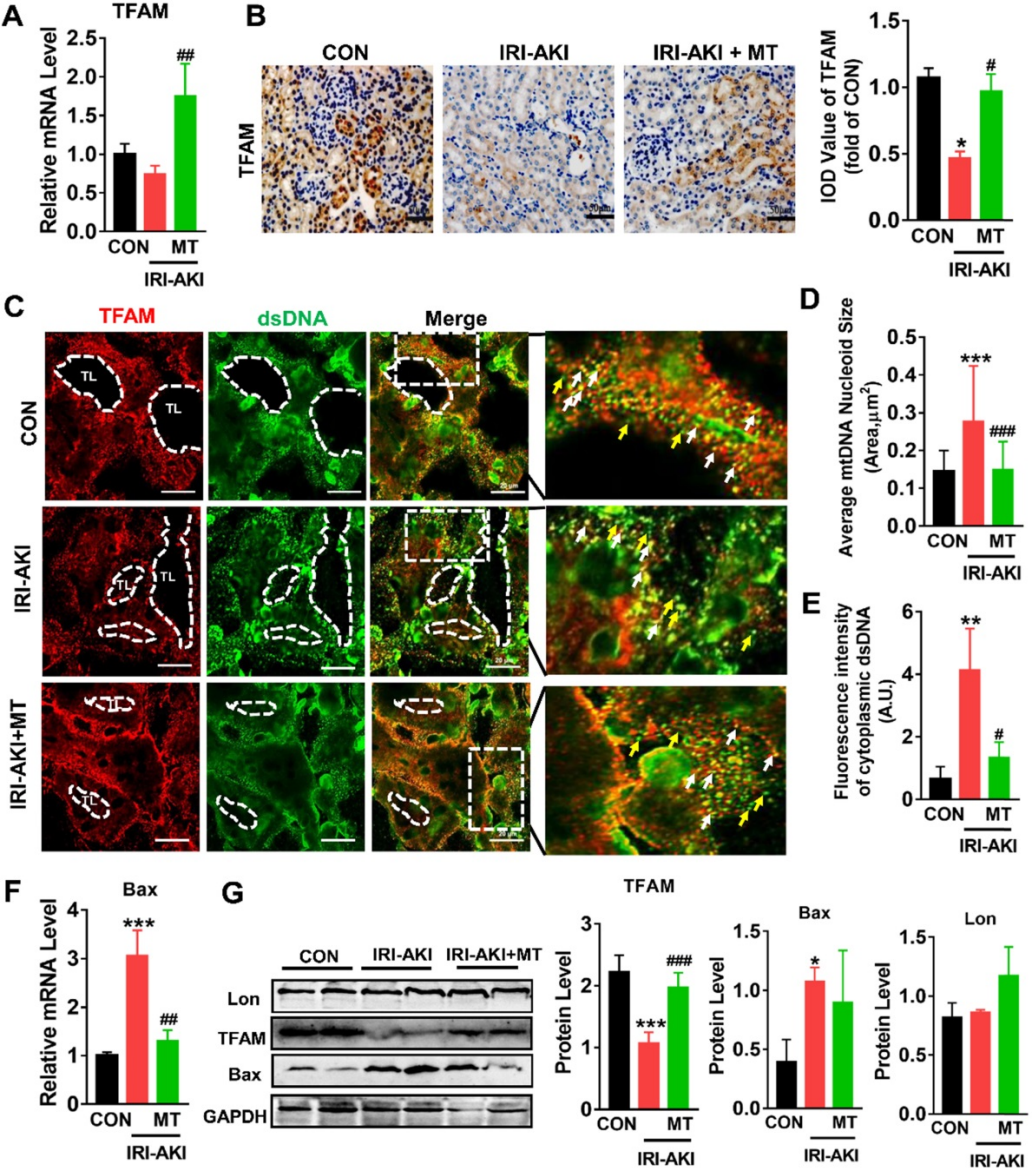
**mtROS-induced TFAM deletion and mtDNA instability in the kidneys of IRI-AKI mice.** (A) Real-time PCR analysis of TFAM mRNA levels in the kidneys of mice on day 5 after IRI-AKI (n = 6; *p < 0.05 *vs.* CON group; **^##^***p* < 0.01 *vs.* IRI-AKI group). (B) Representative micrographs showing TFAM IHC staining in the kidneys of mice on day 5 after IRI-AKI (scale bar = 50 µm) and quantitative analysis of TFAM intensity. (C) Double-IF staining of TFAM (red) and dsDNA (green) in the tubules (TL) of mice on day 5 after IRI-AKI (scale bar = 20 µm). (D) Average size of mtDNA nucleoids in the kidneys detected by IF staining (n = 15; ***p < 0.001 *vs.* CON group; **^###^***p* < 0.0 *vs.* IRI-AKI group). (E) Quantification of cytoplasmic dsDNA intensity (yellow arrows) (n = 6; **p < 0.01 *vs.* CON group; **^#^***p* < 0.05 *vs.* IRI-AKI group). (F) Real-time PCR analysis of Bax mRNA levels in the kidneys of mice on day 5 after IRI-AKI (n = 6; ***p < 0.001 *vs.* CON group; **^##^***p* < 0.01 *vs.* IRI-AKI group). (G) Western blotting of TFAM, Lon, and Bax proteins in the kidneys of mice on day 5 after IRI-AKI and quantitative analysis of protein expression (n = 6; *p < 0.05 *vs.* CON group; ***p < 0.001 *vs.* CON group; **^###^***p* < 0.001 *vs.* IRI-AKI group).

Consistent with the *in vitro* results, we found that TFAM was mainly expressed in renal tubules and that the mRNA and protein levels of TFAM in the kidneys of IRI-AKI mice were significantly reduced (Figure 8A-B). In the tubules of the control mice, the DAPI signals fully colocalized with the nuclear DNA, and the TFAM signals consistently colocalized with cytosolic DNA (Figure S9). However, the percentage of nucleoids with aberrant mtDNA packaging increased in the renal tubules of the IRI-AKI mice. Moreover, higher levels of leaked mtDNA (dsDNA without TFAM colocalization, indicated by the yellow arrows in Figure 8C and E) and Bax expression (Figure 8F) were found in the kidneys of IRI-AKI mice than in the kidneys of control mice. MT treatment reversed the decrease in TFAM expression in the renal tubules of IRI-AKI mice (Figure 8A-B) and reduced mtDNA instability (Figure 8C-E) in the kidneys of IRI-AKI mice. Moreover, the upregulation of Bax protein and the decrease in TFAM protein in the kidneys of IRI-AKI mice were partially reversed by MT treatment (Figure 8G). However, there was no difference in the Lon protein levels in the different groups (Figure 8G). Again, treatment with vehicle alone did not influence TFAM expression or mtDNA copy number in the kidneys of IRI-AKI mice (Figure S1A-C).

### TFAM deficiency and mtDNA damage in the kidneys of AKI patients

**Figure 9:**
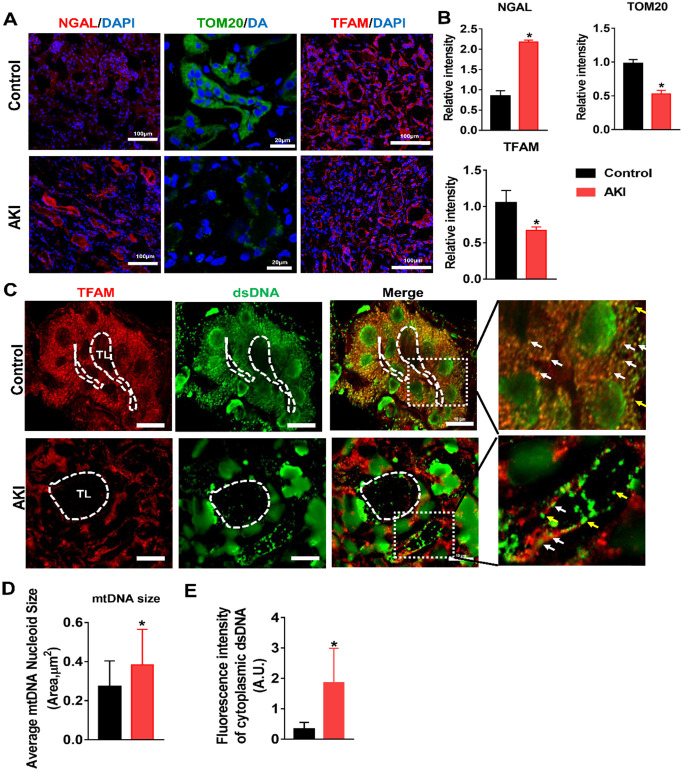
**TFAM deficiency and mtDNA instability in the kidneys of AKI patients.** (A) Representative micrographs showing IF staining of NGAL (scale bar = 100 µm), TOM20 (scale bar = 20 µm), and TFAM (scale bar = 100 µm) in renal sections from AKI patients. (B) Quantitative analysis of NGAL, TOM20, and TFAM expression detected by IF staining. (n = 3; *p < 0.05 *vs.* Control group). (C) Double-IF staining of TFAM (red) and dsDNA (green) in the tubules (TL) of AKI patients (scale bar = 10 µm). (D) Average size of mtDNA nucleoids in the renal tubules detected by IF staining (n = 17; *p < 0.05 *vs.* Control group). (E) Quantification of cytoplasmic dsDNA (yellow arrows) in the tubules by IF staining (n = 6; *p < 0.05 *vs.* Control group).

To validate our findings in HK2 cells and in the mouse IRI-AKI model, changes in TFAM and mtDNA nucleoids were analyzed in renal biopsies from AKI patients. Renal sections from AKI patients exhibited higher levels of NGAL in the tubules, and their renal TFAM and TOM20 protein levels were lower than those of controls (Figure 9A-B). Moreover, we observed enlarged mtDNA nucleoids in the kidneys of AKI patients (Figure 9C-D). The amount of leaked mtDNA (dsDNA without TFAM colocalization, indicated by the yellow arrows) in the kidneys of AKI patients was also higher than that in the controls (Figure 9C and E). These results confirmed that TFAM loss occurs with mtDNA damage in the kidneys of AKI patients.

## Discussion

Mitochondria are essential regulators of cellular energy metabolism and of the cellular redox balance in response to stimuli. Kidneys are organs with a high energy demand, and renal tubules are densely packed with mitochondria 16. Mitochondrial damage, as indicated by elevated mtROS levels and mtDNA depletion, was markedly increased during IRI-AKI, and these lesions were associated with the severity of renal inflammation and renal dysfunction 16. Persistent mitochondrial injury after AKI has also been linked to chronic inflammation, progressive renal fibrosis, and the development of CKD 36,37. Although excessive mtROS have been shown to disturb multiple pathways involved in calcium homeostasis, mitochondrial permeability, and cytochrome C release and to directly induce renal cell death 3,38,39, their specific roles in mitochondrial damage and inflammation during IRI-AKI are not completely understood.

In this study, an initial dose of MT was directly injected into the kidneys of IRI-AKI mice in the early phase after IRI to specifically inhibit the mtROS burst in the kidney. We found that inhibition of mtROS at the early phase was sufficient to attenuate renal dysfunction, cytokine expression, and macrophage infiltration in the kidneys of IRI-AKI mice. Moreover, inhibition of mtROS restored the renal mtDNA level, mitochondrial mass, and ATP production after IRI-AKI. In IRI-AKI mice, some parameters, such as the levels of mtROS, ATP, and mtDNA, were markedly restored by MT therapy, while other indicators of mitochondrial function, such as PGC-1α and ATP5a-1 levels and the mitochondrial length/width ratio, were partially recovered. These results suggest that mtROS are a driving factor in the renal mitochondrial damage and inflammation that occurs in IRI-AKI and that elimination of excessive mtROS at the early phase after IRI would result in superior therapeutic outcomes. To validate the *in vivo* findings, we established ROS-induced renal TEC injury models and evaluated the impact of mtROS on mitochondria and inflammation *in vitro*. Consistent with the *in vivo* data, mtROS inhibition reduced the level of mtDNA loss, mitochondrial respiratory defects, cytokine expression, and macrophage chemotaxis in TECs exposed to stress conditions such as those caused by t-BHP and H/R. These results demonstrate that mtROS are one of the major causative factors of renal mitochondrial damage and inflammation and that they contribute to the pathology of IRI-AKI.

Although the adverse effect of mtROS on renal function in IRI-AKI was previously known, the detailed mechanism has not been fully elucidated. Considering the mtDNA depletion and decreased mitochondrial mass observed after IRI-AKI, we speculated that these renal lesions may be due to disordered mtDNA replication and maintenance. mtDNA is inherited as a protein-DNA complex (the nucleoid), and TFAM serves as the primary coating and packaging protein for mtDNA 40. TFAM binds to mtDNA specifically at promoter regions to activate mitochondrial transcription and nonspecifically throughout the mitochondrial genome to regulate DNA interactions. Interestingly, a decrease in TFAM has been observed in kidneys with AKI and in TECs under oxidative stress 41,42. Consistent with these reports, we found that the amount of TFAM, particularly that in mitochondria, was reduced in TECs with increased ROS and in the renal tubules of IRI-AKI mice. In addition to mitochondria, other sources or types of ROS such as Nox4 43, hydrogen peroxide 44, and iNOS-derived reactive nitrogen species (RNS) 45 have been shown to be involved in the pathology of AKI. Moreover, by using specific inhibitors, we further showed that mtROS might play a predominant role in decreasing TFAM levels and mitochondrial mass in TECs. Mitochondria are highly dynamic organelles, and mitochondrial mass under conditions of stress is regulated by a balance between mitochondrial biogenesis (*e.g.*, TFAM, mtDNA) and degradation (*e.g.*, autophagy) 46. Interestingly, we found that APO treatment partially restored TFAM levels but not TOM20 levels during t-BHP treatment, which might be due to the direct antioxidant effects 47 of APO and/or to its regulatory effects on autophagy 48,49. Our results also suggest that the pathology of ROS-induced renal mitochondrial damage is complicated and that other mechanisms such as autophagy may also be involved. However, in the current study, we focused primarily on the impact of ROS on the TFAM pathway and mtDNA maintenance; the exact mechanism requires exploration in future studies.

TFAM is a nuclear-encoded protein that is synthesized in the cytoplasm, imported into mitochondria, and removed by Lon-mediated degradation 26,40. Thus, the level of intracellular TFAM is determined by a dynamic balance between the transcription and degradation of TFAM. We found that the expression of TFAM mRNA at the transcriptional level was downregulated by mtROS and that it could be rescued by MT. Interestingly, there was a large recovery in TFAM mRNA levels but only a mild recovery in TFAM protein levels in injured HK2 cells after MT treatment, suggesting that mechanisms other than TFAM transcription are also involved. Maintaining an adequate physiological mtDNA copy number is crucial for cellular homeostasis 50. Intracellular TFAM protein level and mtDNA copy number are regulated by posttranslational mechanisms as well as by TFAM transcription 50. Next, we sought to determine whether TFAM degradation is affected by mtROS. Lon is a mitochondrial protease that also binds to specific regions of mtDNA and can eliminate disordered mitochondrial proteins. Lon selectively degrades TFAM protein and impacts mtDNA copy number at the posttranslational level 51. It has been reported that Lon protein is induced in response to various stressors such as hypoxia, ischemia, and heat shock 52. However, we found that the level of Lon protein was unaffected by t-BHP and H/R, whereas its protease activity increased markedly in response to mtROS. In contrast, inhibition of Lon activity by a chemical inhibitor or by siRNA partially reversed the decrease in TFAM and mtDNA copy number in TECs under stress conditions, again confirming that TFAM is a target of Lon protease. This phenomenon (i.e., increased Lon activity but unchanged Lon protein level) may be a result of the variable expression pattern of Lon in different tissues, since our results are based on TECs, while the previous findings were based on other cell types 52. Taken together, these results demonstrate that mtROS can suppress the expression of TFAM protein in TECs by inhibiting its transcription and promoting its Lon-mediated degradation.

Since TFAM directly regulates mtDNA abundance, it plays essential roles in the maintenance of mtDNA stability and mitochondrial biogenesis as well as in the responses of cells to energy demand and the responses of signaling pathways to stimuli 53,54. It has been reported that TFAM and mtDNA stabilize each other by binding to each other; thus, mtDNA can only be stably maintained in the form of nucleoid structures within the mitochondria, whereas both naked DNA and free TFAM in mitochondria are unstable and can be rapidly degraded 34. During disease development, decreased TFAM levels may impair the interaction of TFAM with mtDNA and thus reduce the number of nucleoids and cause the formation of aberrant clusters; this, in turn, results in depletion of mtDNA and inhibition of mitochondrial transcription 55,56. Indeed, we found that TFAM knockdown alone was sufficient to induce mtROS production, aberrant packaging of mtDNA nucleoids, and mitochondrial respiratory defects in TECs. Moreover, the protective effects of MT on mtDNA nucleoids and mitochondrial respiration in TECs under oxidative stress were abolished when TFAM was silenced. These results suggest that TFAM is an essential factor in maintaining mtDNA stability and mitochondrial biogenesis in the kidney. Therefore, the loss of TFAM after IRI-AKI caused a reduction in mtDNA synthesis and nucleoid stability, resulting in impaired mitochondrial energy metabolism and renal function.

Moreover, we found that inhibition of mtROS also attenuated the inflammatory response (*e.g.*, cytokine release and infiltration by macrophages) in the kidneys of IRI-AKI mice and in TECs maintained under stress conditions. After renal damage, the release of endogenous damage-associated molecular patterns (DAMPs) such as ROS and mtDNA fragments by injured cells can activate cellular receptors, leading to downstream inflammation in the kidneys 57. ROS have been proposed as one major type of DAMP that induces renal inflammation in animal and cell models of AKI 37. Interestingly, we found that inhibition of mtROS reduced cytokine release and macrophage chemotaxis in TECs under stress conditions but that these rescue effects were abolished when TFAM was knocked down, suggesting that other mechanisms are also involved. In AKI patients, the amount of urinary mtDNA was elevated and positively correlated with the severity of renal injury 32. Moreover, TFAM deficiency was shown to induce aberrant packaging of nucleoids, thereby promoting leakage of mtDNA into the cytosol and the production of cytokines 58. Consistent with these reports, we found that mtROS increased the number of aberrant mtDNA nucleoids and the amount of leaked mtDNA in the cytosol and that these changes consequently induced cytokine release and macrophage infiltration in TECs and renal tubules. A recent study showed that mitochondrial macropores formed by activated Bax protein under stress allow the release of mtDNA into the cytosol 59. Similarly, we observed activation of Bax protein and the formation of macropore-like structures in TECs in response to mtROS. Furthermore, we also observed decreased amounts of TFAM, together with increased numbers of aberrant nucleoids and increased amounts of leaked mtDNA, in the renal tubules of AKI patients. These results support the hypothesis that mtROS induce mtDNA release and subsequent inflammation of the kidneys by impairing the stability of TFAM and mtDNA.

## Conclusion

**Figure 10:**
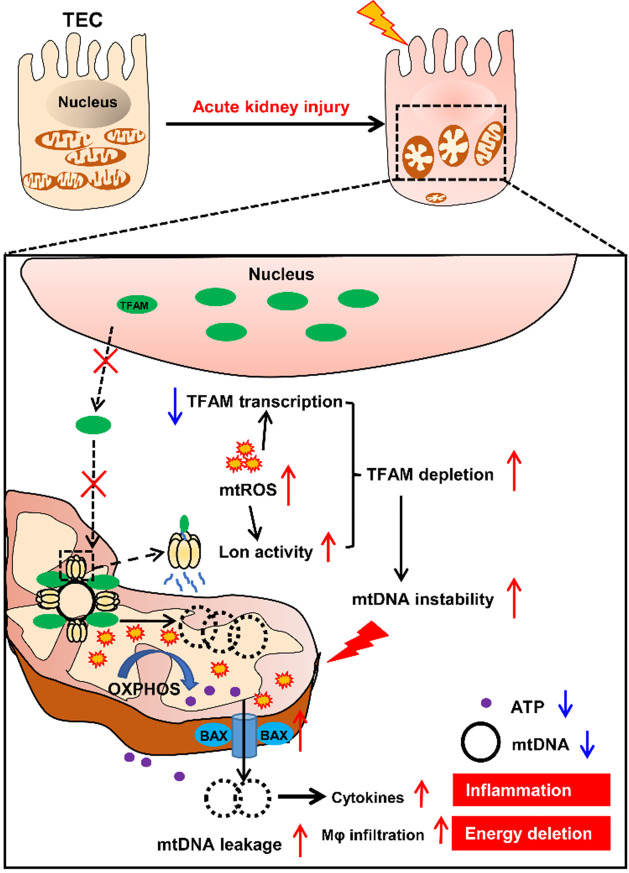
** Schematic diagram of the findings of this study.** The mtROS burst after IRI-AKI reduces TFAM transcription and promotes Lon-mediated TFAM degradation in renal tubular cells (TECs), leading to decreased mitochondrial TFAM levels in these cells. The loss of TFAM triggered by mtROS also causes depletion of mtDNA and impaired mitochondrial energy metabolism as well as elevated cytosolic mtDNA release and enhanced cytokine production in TECs, thereby exacerbating renal damage after IRI-AKI.

In summary, our results demonstrate that mtROS are one of the drivers of mitochondrial dysfunction and inflammation of the renal tubules in IRI-AKI. Mechanistically, mtROS reduced the abundance of TFAM in TECs by inhibiting its transcription and promoting its Lon-mediated degradation. TFAM deficiency further reduced mtDNA synthesis and mitochondrial biogenesis and thus induced mtDNA depletion and mitochondrial respiratory defects in TECs during IRI-AKI. At the same time, the loss of TFAM also impaired the stability of mtDNA nucleoids and promoted the release of mtDNA fragments and cytokine production in TECs during IRI-AKI (Figure 10). Therefore, mtROS-induced TFAM depletion plays an essential role in the pathology of IRI-AKI, and defective TFAM may serve as a therapeutic target for the promotion of renal recovery after IRI-AKI.

## Supplementary Material

Supplementary figures and tables.

## Abbreviations

AKI: acute kidney injury
BUN: blood urea nitrogen
CCK8: cell counting kit 8
CON: control
CREA: serum creatinine
IRI: ischemia/reperfusion injury
NC: negative control
siRNA: si-TFAM
si-Lon: Lon siRNA
ROS: reactive oxygen species
SDS-PAGE: sodium dodecyl sulfate-polyacrylamide gel electrophoresis
TFAM: mitochondrial transcription factor A
MT: Mito-Tempo
mtDNA: mitochondrial DNA
t-BHP: tert-butyl hydroperoxide
mtROS: mitochondrial ROS
TECs: tubule epithelial cells
TUNEL: terminal deoxynucleotidyl transferase mediated dUTP nick-end labeling staining
IF: Immunofluorescence
