# Supplementary material for: Mitochondrial ROS promote mitochondrial dysfunction and inflammation in ischemic acute kidney injury by disrupting TFAM-mediated mtDNA maintenance
Source: Theranostics. 2021 Jan 1;11(4):1845–63. doi: 10.7150/thno.50905 (PMC7778599; doi:10.7150/thno.50905)
Supplement: Supplementary file 1 — Supplementary figures and tables. [file thnov11p1845s1.pdf]

Supplementary Materials

Mitochondrial ROS promote mitochondrial dysfunction and inflammation in ischemic acute kidney injury by disrupting TFAM-mediated mtDNA maintenance

Meng Zhao [a#], Yizhuo Wang [a#], Ling Li [b], Shuyun Liu [a], Chengshi Wang [a], Yujia Yuan [a], Guang Yang [c], Younan Chen [a], Jingqiu Cheng [a], Yanrong Lu [a], Jingping Liu [a]*

[a] Key Laboratory of Transplant Engineering and Immunology, National Clinical Research Center for Geriatrics, Frontiers Science Center for Disease-related Molecular Network, West China Hospital, Sichuan University, Chengdu, China

[b] Department of Nephrology, West China Hospital, Sichuan University, Chengdu, China

[c] Animal Center, West China Hospital, Sichuan University, Chengdu, China.

[#] Co-first authors: These authors contributed equally to the authorship of this article

*Corresponding Authors: Jingping Liu

Address: Key Laboratory of Transplant Engineering and Immunology, West China Hospital, Sichuan University, No. 1 Keyuan 4th Road, Gaopeng Ave, Chengdu 610041, China

Tel: +86-28-85164029, Fax: +86-28-85164030

E-mail: liujingping@scu.edu.cn

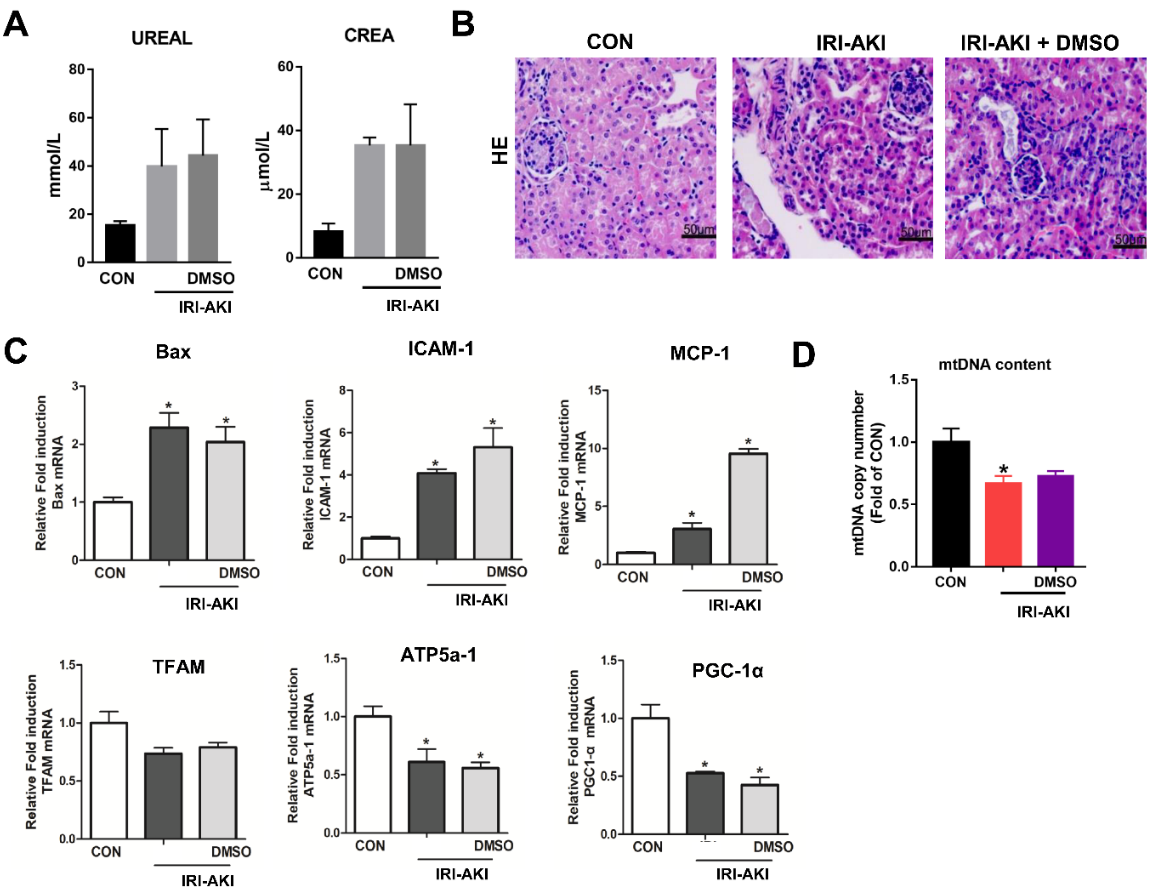

Figure S1. The vehicle (DMSO/PBS) alone had no influence on renal lesions in IRI-AKI mice. (A) Serum CREA and UREAL concentrations of mice on day 5 after IRI-AKI (n = 6; *p < 0.05 *vs.* CON group). (B) Representative micrographs of renal H&E staining in mice with different treatments. (C) Real-time PCR analysis of Bax, ICAM-1, MCP-1, TFAM, ATP5a-1, and PGC-1α mRNA levels in kidneys of mice on day 5 after IRI-AKI (n = 3; *p < 0.05 *vs.* CON group). (D) mtDNA copy number in kidneys of mice on day 5 after IRI-AKI (n = 3; *p < 0.05 *vs.* CON group).

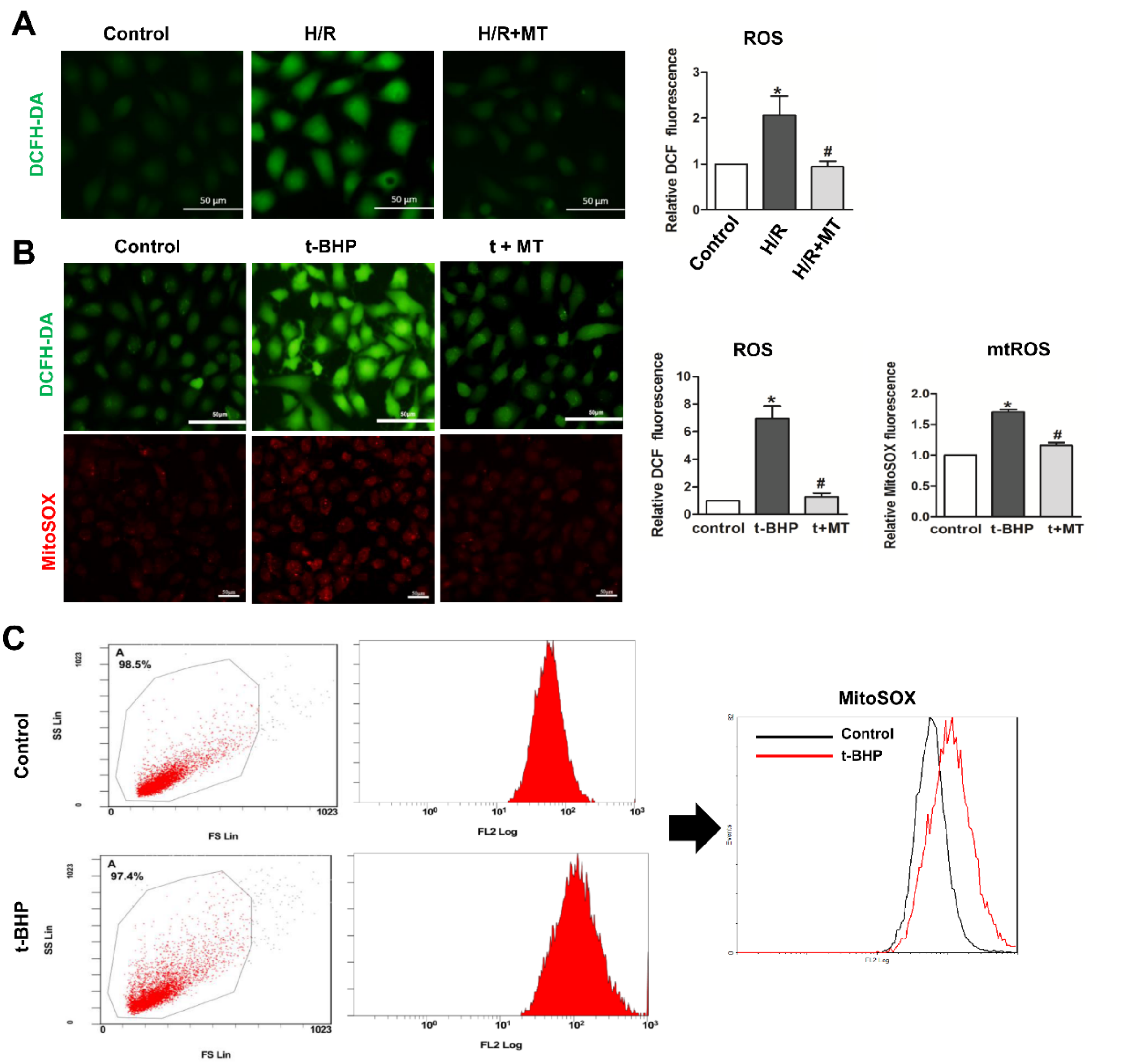

Figure S2. Measurement of ROS production in HK2 cells under different stress conditions. (A) Determination of intracellular ROS in the HK2 cells under H/R conditions using the DCFH-DA staining (scale bar = 50 μm). (B) Determination of intracellular ROS and mtROS in HK2 cells under t-BHP conditions with the DCFH-DA and MitoSOX staining (Scale bar = 50 μm). (C) Determination of mtROS level in HK2 cells under t-BHP conditions using flow cytometry with MitoSOX staining.

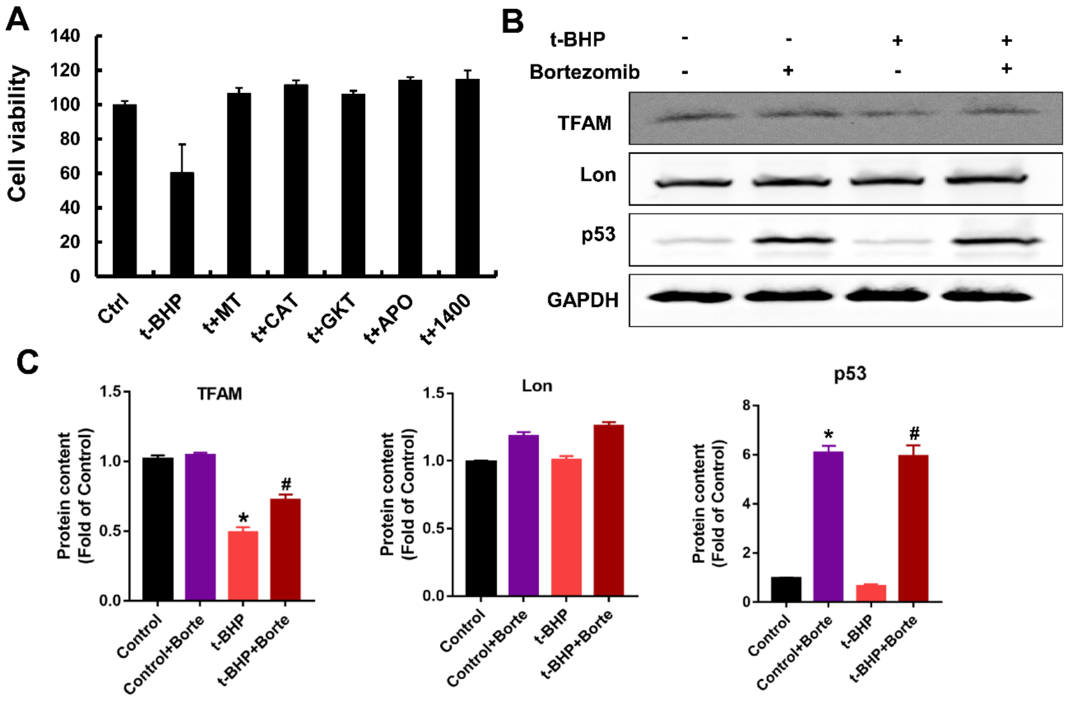

Figure S3. TFAM degradation was enhanced in HK2 cells under oxidative stress. (A) Cell viability was determined by CCK8 assay (n = 3; *$p < 0.05$ *vs.* Control group; $^{\#}p < 0.05$ *vs.* t-BHP group). (B) Western blotting of TFAM, Lon, and p53 proteins in HK2 cells under t-BHP conditions with or without bortezomib treatment. (C) Quantitative analysis of protein expression detected by western blotting (n = 3; *$p < 0.05$ *vs.* Control group; $^{\#}p < 0.05$ *vs.* t-BHP group).

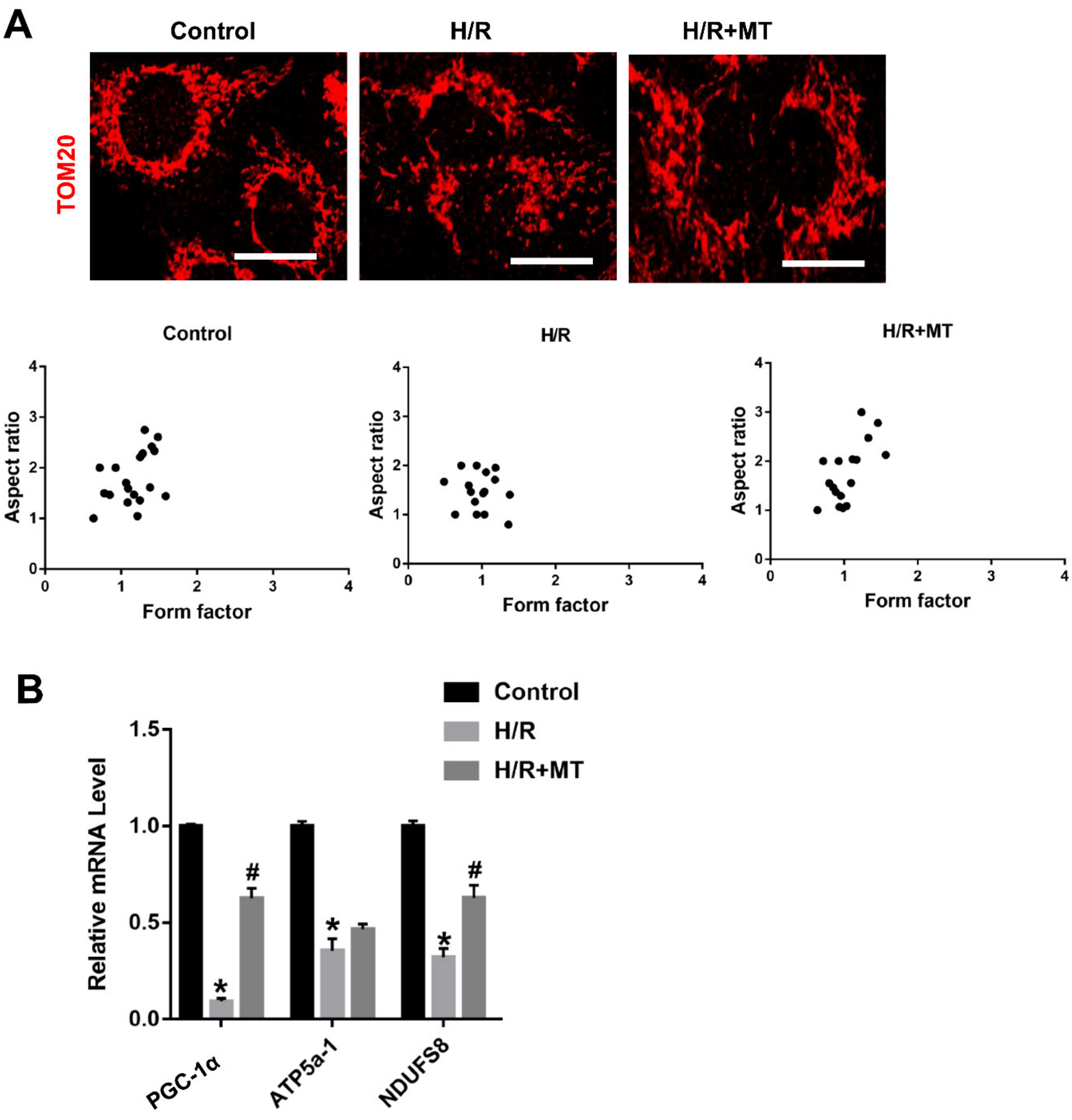

Figure S4. (A) Observation of mitochondria and quantification of mitochondrial length in HK2 cells under H/R conditions. HK2 cells were treated with or without MT and then stained with anti-TOM20. (B) Real-time PCR analysis of PGC-1α, ATP5a-1, and NDUFS8 mRNA levels in HK2 cells under H/R conditions with or without MT treatment (n = 3; *$p < 0.05$ *vs.* Control group; $^{\#}p < 0.05$ *vs.* H/R group).

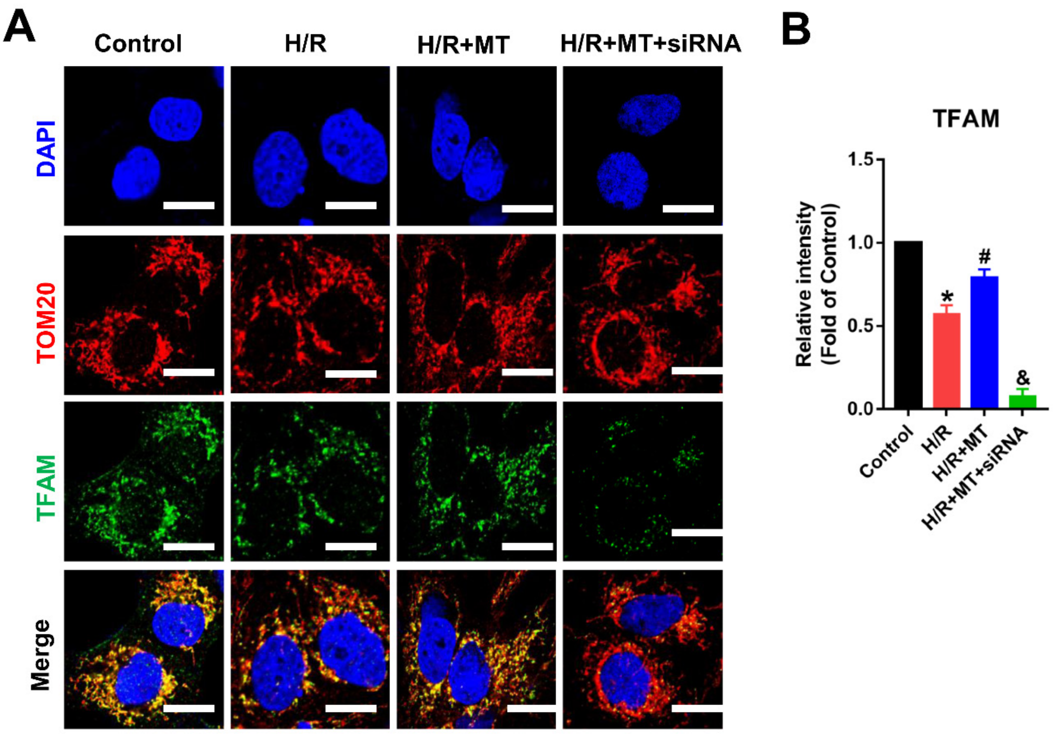

Figure S5. (A) Double-IF staining of TOM20 (red) and TFAM (green) in the HK2 cells under H/R conditions with or without TFAM siRNA treatment (scale = 10 μm). (B) Quantitative analysis of TFAM protein expression in the HK2 cells (n = 6; *$p < 0.05$ *vs.* Control group; $^{\#}p < 0.05$ *vs.* H/R group; $^{\&}p < 0.05$ vs. MT group).

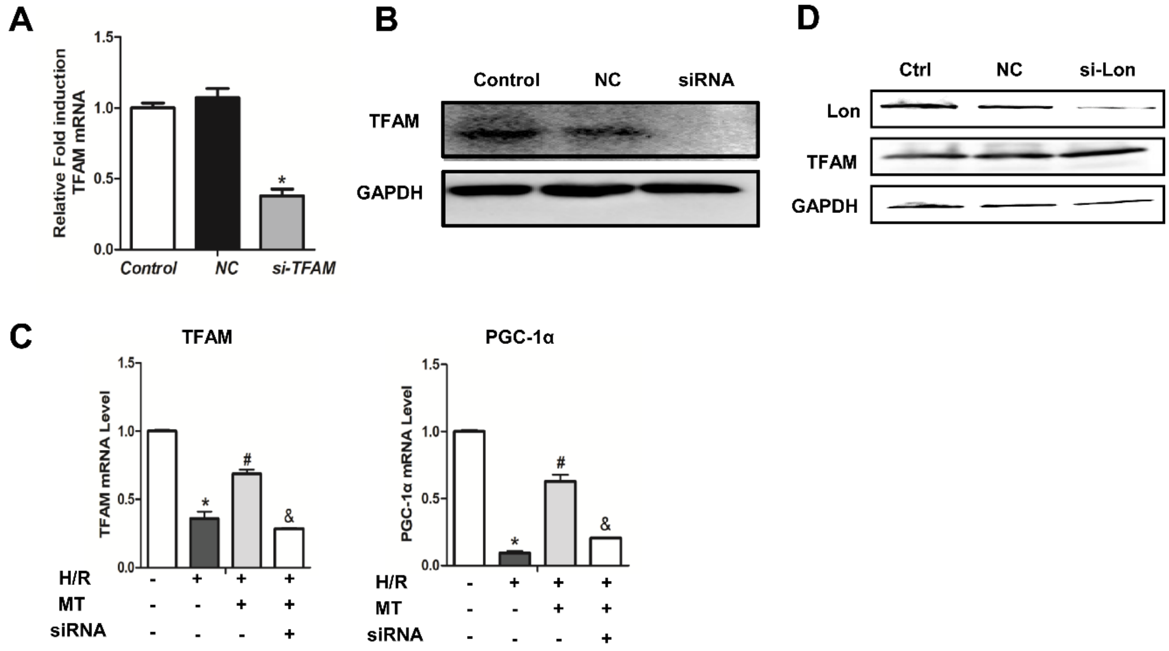

Figure S6. TFAM deficiency abolished the protective role of mtROS scavenger in the HK2 cells. (A) Real-time PCR analysis of TFAM mRNA levels in HK2 cells. (B) Western blotting analysis of TFAM protein levels in the HK2 cells at 48 h after transfection. (C) Real-time PCR analysis of TFAM and PGC-1α mRNA levels in HK2 cells under H/R conditions (n = 3; *p < 0.05 *vs*. Control group; #p < 0.05 *vs*. H/R group; &P < 0.05 *vs*. MT group).

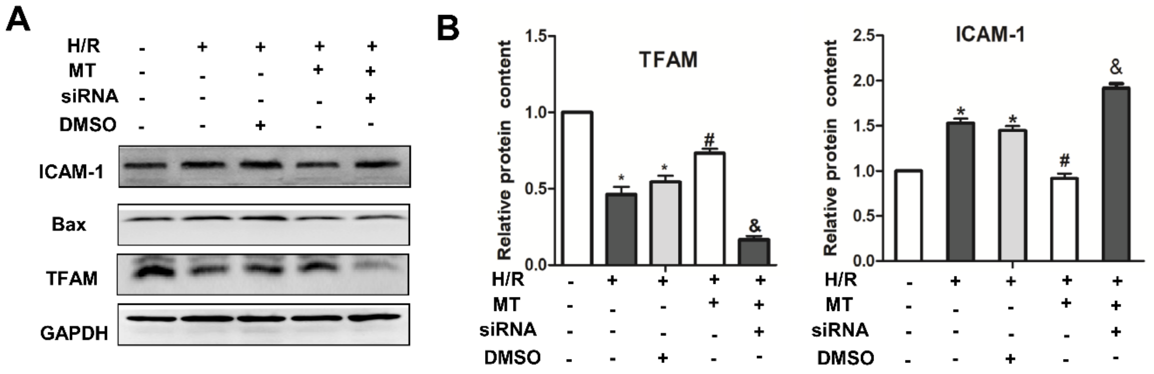

Figure S7. Loss of TFAM under oxidative stress induced cytokine production in HK2 cells. (A) Western blotting analysis of ICAM-1, Bax, and TFAM protein levels in HK2 cells under H/R conditions with various treatments. (B) Quantitative analysis of ICAM-1 and TFAM protein expression detected by western blotting (n = 3; *$p < 0.05$ *vs.* Control group; $^{\#}p < 0.05$ *vs.* H/R group; $^{\&}P < 0.05$ *vs.* MT group).

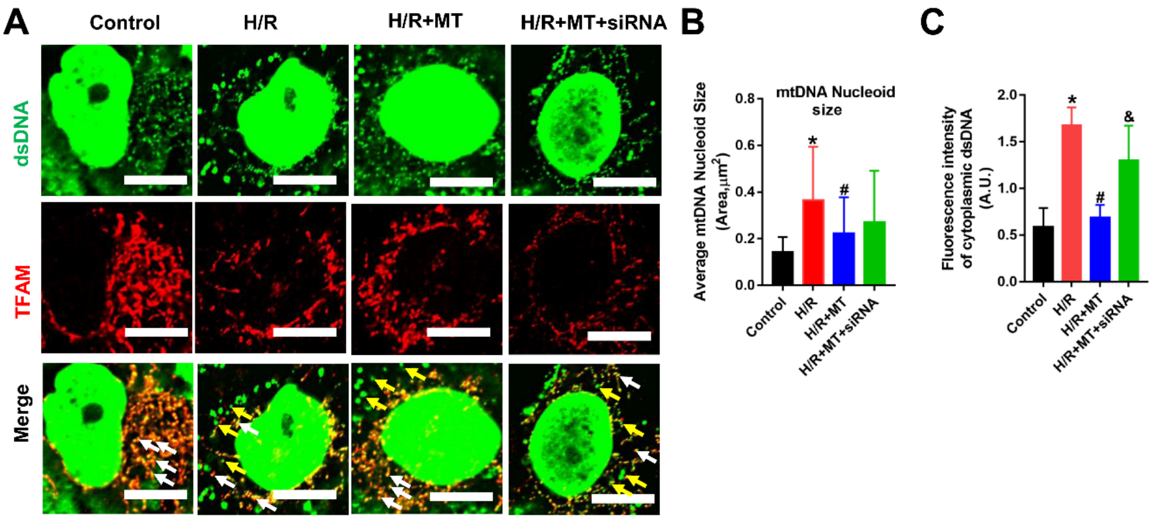

Figure S8. (A) Double-IF staining of TFAM (red) and dsDNA (green) in HK2 cells under H/R conditions with various treatments (scale bar = 10 μm). (B) Average size of mtDNA nucleoid in HK2 cells of different groups (n = 20 cells; $^{*}p < 0.05$ *vs.* Control group; $^{\#}p < 0.05$ *vs.* H/R group). (C) Quantification of cytoplasmic dsDNA (yellow arrows) intensity in HK2 cells (n = 16 cells; $^{*}p < 0.05$ *vs.* Control group; $^{\#}p < 0.05$ *vs.* H/R group; $^{\&}p < 0.05$ vs. MT group).

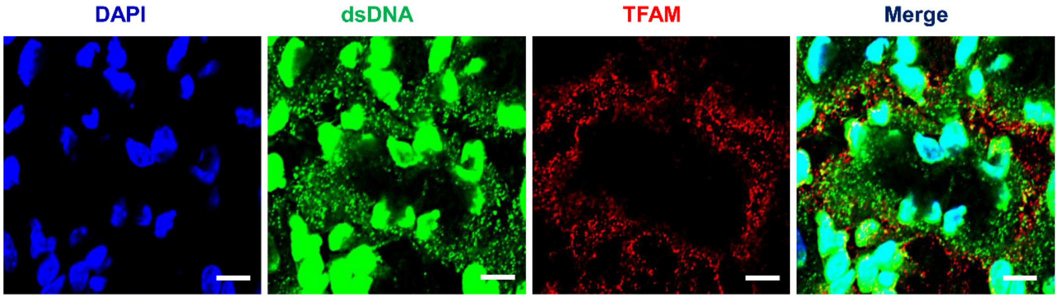

Figure S9. Representative images of the TFAM (red), dsDNA (green), and DAPI costaining in kidney of normal control mice (scale bar = 10 μm).

Table S1 Primers used in Real-time PCR assay

Gene	Sequence 5'-3'	Species
TFAM	AGCTCAGAACCCAGATGCAA	
	CCGCCCTATAAGCATCTTGA	
PGC1-α	TGCTGAAGAGGCAAGAGACA	
	CACACACGCACACTCCATC	
NDUFS8	CATCTACTGCGGCTTCTGC	
	GGGCGTCACCGATACAAGT	
ATP5a-1	AGAGGACAGGAGCCATTGTG	
	TCAGACCAACTCGCCTACG	
UQCRC1	CAGTCCTCTCAGCCCACTTG	Human
	CCGATTCTTTGTTCCCTTGA	
IL-1β	TGGCAGAAAGGGAACAGAAA	
	CTGGCTGATGGACAGGAGAT	
TNF-α	TGCTGCACTTTGGAGTGATCG	
	TGTCACTCGGGGTTCGAGAAG	
GAPDH	ACCACAGTCCATGCCATCAC	
	TCCACCACCCTGTTGCTGTA	
TFAM	CACCCAGATGCAAAACTTTCAG	
	CTGCTCTTTATACTTGCTCACAG	
PGC1-α	CACCAAACCCACAGAAAACAG	
	GGGTCAGAGGAAGAGATAAAGTTG	
ATP5a-1	CATTGGTGATGGTATTGCGC	
	TCCCAAACACGACAACTCC	
Bax	TGGAGATGAACTGGACAGCA	Mouse
	TGAAGTTGCCATCAGCAAAC	
MCP-1	AGTTGACCCGTAAATCTGAAGC	
	GTGGTTGTGGAAAAGGTAGTGG	
ICAM-1	ACCCAACTGGAAGCTGTTTG	
	CACACTCTCCGGAAACGAAT	
TNF-α	CCAGGAGAAAGTCAGCCTCCT	
	TCATACCAGGGCTTGAGCTCA	
GAPDH	CAGATCCACAACGGATATATTGGG	
	CATGACAACTTTGGCATTGTGG	

Table S2 Primers used in mtDNA copy number assay

Gene	Sequence 5'-3'	Species
hB2M	TGTTCCTGCTGGGTAGCTCT	Human
	CCTCCATGATGCTGCTTACA	
mtND1	CACTTTCCACACAGACATCA	
	TGGTTAGGCTGGTGTTAGGG	
COX2	ATAACCGAGTCGTTCTGCCAAT	Mouse
	TTTCAGAGCATTGGCCATAGAA	
Rsp18	TGTGTTAGGGGACTGGTGGACA	
	CATCACCCACTTACCCCCAAAA	
